# A Review of Acoustic Impedance Matching Techniques for Piezoelectric Sensors and Transducers

**DOI:** 10.3390/s20144051

**Published:** 2020-07-21

**Authors:** Vivek T. Rathod

**Affiliations:** Department of Electrical and Computer Engineering, Michigan State University, East Lansing, MI 48824, USA; rathodvi@msu.edu

**Keywords:** acoustic impedance, ultrasonic transducer, matching layer, piezoelectric sensor, biomedical imaging, nondestructive evaluation, structural health monitoring, acoustic emission, energy harvesting

## Abstract

The coupling of waves between the piezoelectric generators, detectors, and propagating media is challenging due to mismatch in the acoustic properties. The mismatch leads to the reverberation of waves within the transducer, heating, low signal-to-noise ratio, and signal distortion. Acoustic impedance matching increases the coupling largely. This article presents standard methods to match the acoustic impedance of the piezoelectric sensors, actuators, and transducers with the surrounding wave propagation media. Acoustic matching methods utilizing active and passive materials have been discussed. Special materials such as nanocomposites, metamaterials, and metasurfaces as emerging materials have been presented. Emphasis is placed throughout the article to differentiate the difference between electric and acoustic impedance matching and the relation between the two. Comparison of various techniques is made with the discussion on capabilities, advantages, and disadvantages. Acoustic impedance matching for specific and uncommon applications has also been covered.

## 1. Introduction

Piezoelectric materials serve with a wide variety of applications when used in many ways. As bulk materials, they fulfill the purpose of actuation and sensing in macro and large devices. Some applications of bulk piezoelectric materials are biomedical ultrasound [1,2,3], piezoelectric–catalysis-based environmental remediation [4], structural control [5,6,7], vibration control [8,9], vibration sensing [10,11], and structural health monitoring [12,13,14]. As thin films ranging from few micrometers to nanometers, they have seen growing use in applications such as force microscopy [15], nano-positioning [16], micromechanical systems (MEMS) [17,18,19] and nanoelectromechanical systems (NEMS) [20,21], energy harvesters [22,23], etc. Some well-known applications of thin films are MEMS microphone [24], headphone [24], loudspeaker [24,25,26], acoustic emission sensor [27,28], vibration sensor [10,14], inertial sensor [19], tactile sensors [29,30,31], power harvesting [32,33], ultrasound transducers [34,35,36,37,38], and guided wave sensors [39,40,41]. Thin films are preferably made of nontoxic piezopolymers or piezocomposites. From the past two decades, major improvements in the piezoelectric material properties and manufacturing techniques have reduced the manufacturing cost [42,43,44,45,46,47]. The low cost and excellent properties have led to the surge in their use as materials for the internet of things (IOT) applications [48,49]. The above-mentioned applications involve the operation at quasi-static, infrasonic, audio, and ultrasonic frequencies [50]. The development of high-frequency ultrasonic imaging has pushed the limits of these technologies, resulting in diagnostic capability with microscopic information of living tissue involving eye, skin, and vascular muscles [51,52]. Therefore, piezoelectric materials are used most widely in biomedical applications [53] such as ultrasonography [54,55,56,57,58,59,60,61,62], due to the ability to produce real-time high resolution 3D images of biological tissue, eliminating the need for hazardous X-rays. Special applications such as scanning acoustic microscopy, cellular stimulation, and particle manipulation are the results of very high-frequency ultrasound ≥100 MHz [63,64,65,66].

**Figure 1:**
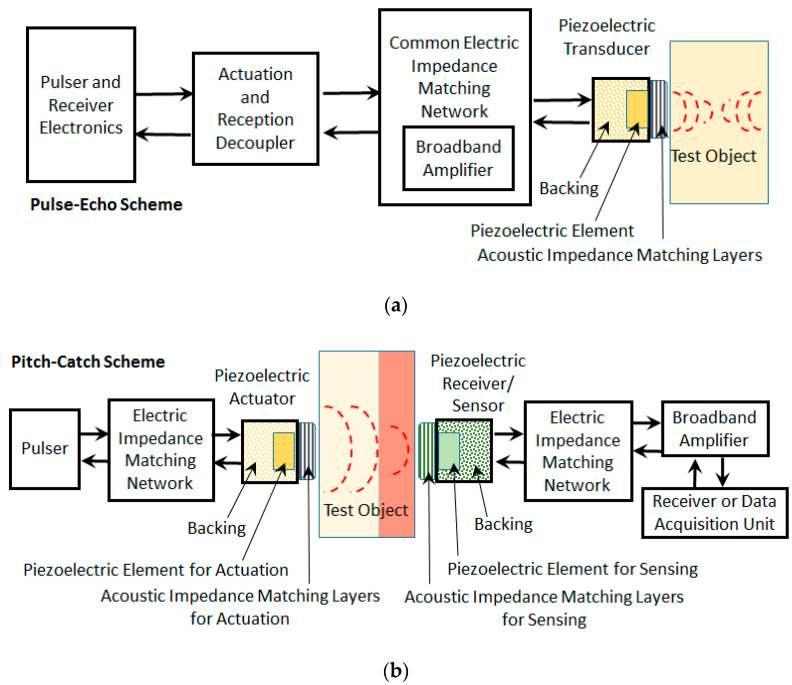
Schematic of elements involved in the acoustic and electric impedance matching (**a**) for a pulse–echo mode where the same piezoelectric element is used both as actuator and sensor i.e., transducer with same acoustic matching layers, backing material, and electric impedance matching network, and (**b**) for a pitch–catch mode where the different piezoelectric elements are used as an actuator and sensor with different electric impedance matching networks, backing material, and acoustic matching layers.

The efficient performance of piezoelectric devices depends on the proper matching of electric and acoustic impedances, especially when considered in their design stages. With a rising development of piezoelectric devices, there is a need for versatile tools to assist with the electric and acoustic impedance matching, especially on a common platform. However, the requirement of broad interdisciplinary knowledge from a variety of disciplines such as acoustics, vibration, electrical, electronics, signal processing, imaging, anatomy, physiology, materials, etc., has made the design procedures available so far mostly empirical. The design and manufacturing procedures of highly efficient transducers and sensors remain with most manufacturers as trade secrets of the highest order [67]. A word of caution is needless to distinguish electric impedance from mechanical or acoustic impedance, since these two serve different purposes but are dependent on each other. Figure 1 shows a schematic of a pitch–catch and pulse–echo system with details of the transducer with acoustic impedance matching layers and its associated electronics.

**Figure 2:**
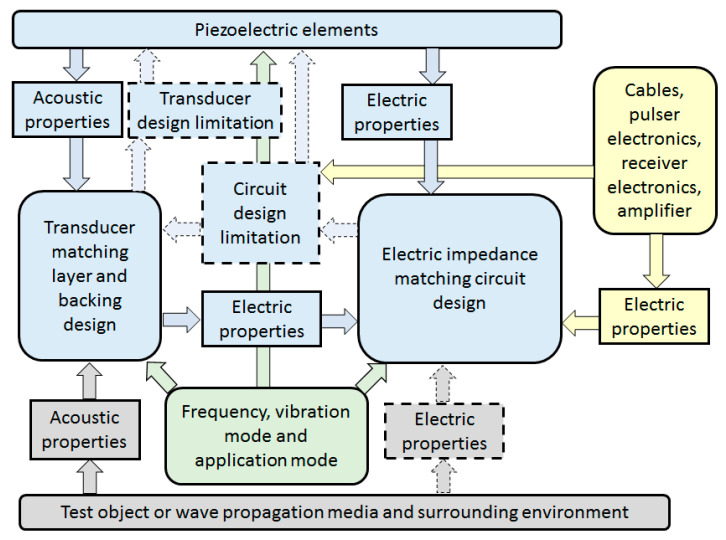
Schematic showing the process flow of electric and acoustic impedance matching involved in the design of piezoelectric receiver or transducer systems. The design elements indicated by dotted lines are uncommon and are used in the design of highly advanced transducers (such as very high frequency, high signal-to-noise ratio (SNR) and low-power considerations).

Figure 2 shows the process flow of electric and acoustic impedance matching while designing a piezoelectric transducer system. Electric impedance matching enables an efficient transfer of electric power whereas acoustic matching enables the proper transfer of acoustic energy. Electrical impedance matching involves the design of the electric matching circuit that connects the driving or receiving circuit to the transducer. It also involves the design of the geometry and electric property of the cable and transducer. Acoustic impedance matching involves the selection of materials and geometric design of the matching layer, backing layer, and the piezoelectric element. The passive matching techniques involves the matching of acoustic passive layers with the piezoelectric element, whereas the active matching technique involves the modification of piezoelectric element properties, eliminating the need for matching layers. A change of geometry during acoustic matching may change the electric property, which leads to redesigning of the electric impedance matching network. Thus, both electric and acoustic impedance matching involves the transducer design considerations in common, especially the piezoelectric element, which is directly related. Thus, highly optimized design can only result when both electric and acoustic impedance matching are considered together. Such an optimized design is possible using commercial software and an iterative design procedure.

Often, the transducer and driving/receiver circuit available commercially have fixed specifications, which cannot be altered. In addition, components available commercially for the design of electric impedance matching circuit are difficult to know before the design of the transducer. Thus, for the most uncommon uses in the laboratory, limited uses in industries, and for special applications, the transducer is designed first followed by its electric impedance matching with source and receiver [68]. The acoustic impedance matching of a transducer involves the matching of piezoelectric element properties with the media in which the acoustic signal is being transmitted. The design is carried out for a given operating frequency or for a given frequency range with possible information of wave propagation media (air, water, biological tissue etc.) and operating environment (temperature, corrosion, radiation etc.) [69].

High-performance transducers involve a design process where choosing active materials with high electromechanical coupling factor is one of the easiest ways. Another option is an improvement in the acoustic design including both matching layers and the backing. Apart from acoustic matching, the backing material is designed to achieve proper isolation to minimize reflection within the piezoelectric element and transducer casing, as shown in Figure 1. Based on the processing method and poling direction, the acoustic and piezoelectric properties of the piezoelectric materials can differ, which has to be considered while designing the acoustic impedance matching layers. In addition, there are limitations on the way of using the piezoelectric material depending upon the polarization direction, anisotropy of elastic constants, and electrical and thermal considerations. In highly efficient designs especially for the pitch–catch type of operation, the exciter (actuator) and receiver (sensor) are designed with a different type of piezoelectric elements for which different matching layers and backing material are needed. In advanced transducer designs, the material properties of the piezoelectric element are modified to assist with the acoustic impedance matching. The transducer design process is complicated due to various factors influencing the design ranging from application needs to the limitations posed by the availability of material and hardware components for fabrication. Thus, a common tool is unavailable to design acoustic and electric impedance matching for the transducer system together.

A detailed review of electric impedance matching techniques for piezoelectric materials used in the configuration of sensors, actuators, and transducers has been provided in Ref. [70]. In this paper, the focus has been laid on the acoustic impedance matching techniques for piezoelectric devices. Depending on the requirements of an application and limitations arising due to the design constraints of the electric and acoustic elements of the transducer system, the acoustic impedance matching procedure may be dependent or independent. The independent design involves the matching of acoustic impedance of piezoelectric element(s) to the wave propagating medium. The dependent procedure involves iterative process or trials where the matching layers are redesigned to achieve optimal performance for varying the acoustic and electric configuration of the piezoelectric element [71]. Each iteration of the dependent procedure is like an independent procedure. Many techniques have been reported so far to match the acoustic impedance of piezoelectric element with the wave propagating medium. These techniques involve changing the geometry or material property of the matching layer or the piezoelectric element itself (for example, piezocomposites with varying filler or fiber content).

Section 1 provides a brief review of common piezoelectric and acoustic matching layer materials used for acoustic impedance matching. Section 2 presents the available tools to assist in the various stages of acoustic impedance matching. Special materials that are different from the conventional materials developed for acoustic impedance matching have been discussed in Section 3. Section 4 discusses the acoustic impedance matching techniques developed for specific types of transducers. Section 5 discusses the acoustic impedance matching techniques that consider requirements arising from specific applications. Section 6 presents the concluding remarks.

### 1.1. Theory of Piezoelectricity and Vibration Modes

**Figure 3:**
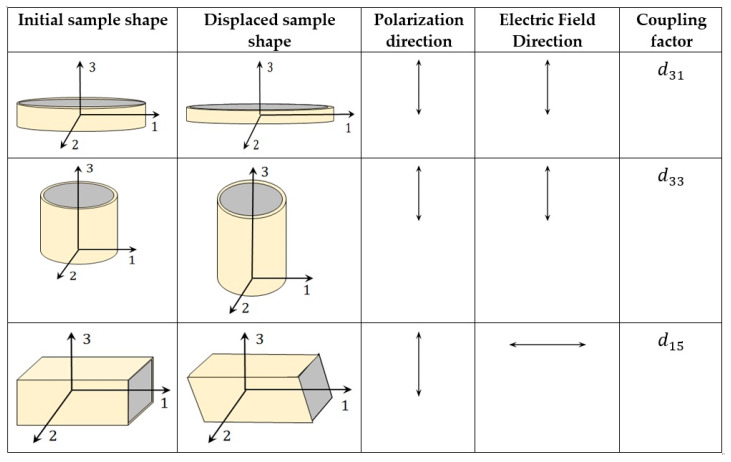
Typical vibration modes and corresponding piezoelectric coupling factors [75].

Most of the transducers used in biomedical, nondestructive evaluation (NDE), and structural health monitoring (SHM) applications operate based on the principle of piezoelectricity. Piezoelectricity is a phenomenon where an electrical charge is generated under the application of mechanical stress or vice versa. The elastic and electrical properties in piezoelectric materials are coupled. Therefore, mechanical parameters such as stress σ and strain ε, and the electrical parameters such as electric field E and dielectric displacement D are related by constitutive relations [72]. For an unstressed non-piezoelectric medium, the electrical relation is given by

(1) $$D_{m}=\epsilon_{mn}E_{n}\;,$$

where $\epsilon_{mn}$ is the stress-free permittivity matrix of the piezoelectric medium. The stress-free permittivity of piezoelectric material is obtained by measuring it with stress-free boundary conditions. At zero electric field, the mechanical relation for the same medium is given by

(2) $$\varepsilon_{ij}=S_{ijkl}\sigma_{kl}\;,$$

where $S_{ijkl}$ is the tensor representing the short-circuit mechanical compliances of the medium. The elements of $S_{ijkl}$ are measured using the strain developed for the applied stress with the electrodes of the piezoelectric material being shorted. The shorting of electrodes results in the electrical boundary conditions with zero field across faces but allows the charge to flow from the positive to the negative terminal. Thus, shorting prevents any effect on compliance measurement due to piezoelectricity. The interaction of electrical and mechanical variables in a linear regime is described by the constitutive relations given by

(3) $$D_{m}=d_{mkl}\sigma_{kl}+\epsilon_{mn}E_{n}$$

(4) $$\varepsilon_{ij}=S_{ijkl}\sigma_{kl}+d_{nij}E_{n}\;,$$

where $d_{ijn}$ is a tensor of the piezoelectric material. Equation (3) is called the sensing law, which describes the direct piezoelectric effect and is used to estimate the total charge and thereby the voltage developed by the receiver or sensor or transducer in a sensing configuration. Equation (4) is called the actuation law; it describes the converse piezoelectric effect and is used to estimate the stress and thereby the force developed by an exciter, actuator, or transducer in an actuating configuration. Depending upon the piezoelectric material geometry and poling direction, the above Equations (3) and (4) are applied to determine the outcome parameters such as force or voltage. These parameters are further related to material properties in various ways depending on the modes of operation and application. Therefore, these performance parameters are many and have been reported by researchers and engineers to assist in the design of transducers and their acoustic matching layers. These parameters will be further discussed in this section. Figure 3 shows the commonly used shapes of piezoelectric materials and their vibration modes. Here, poling is along axis 3. Polarization destroys the isotropy in the direction of poling and retains it in any other direction perpendicular to it (i.e., directions along axes 1 and 2). The set of independent piezoelectric and dielectric constants for this symmetry are $d_{31}$, $d_{33}$, $d_{15}$, $\epsilon_{11}$ and $\epsilon_{33}$. The $d_{33}$ coefficient describes the electric polarization generated in the same direction as the stress applied and is called the longitudinal coefficient [72]. The $d_{31}$ coefficient describes the electric polarization generated in a direction perpendicular to the direction of the applied stress and is called the transverse coefficient [11,72]. The property $d_{33}$ is a commonly used property in biomedical ultrasound, NDE probes, vibration control, and energy harvesting applications. The property $d_{31}$ is utilized by SHM, energy harvesting, vibration control, monitoring, etc [11,40,41,73]. The $d_{15}$ shear piezoelectric coefficient describes the polarization generated, which is inclined to the direction of applied stress [74]. 

Although a piezoelectric material can exhibit many piezoelectric coefficients and have many properties, they are selected depending on the desired vibration mode and operating modes. Consider an example for the vibrational mode with a predominant involvement of the thickness. For an application where the thickness or out-of-plane vibration measurement is desired, the piezoelectric element with greater $d_{33}$ property is selected, with $d_{31}$ and $d_{15}$ being ignored.

### 1.2. Piezoelectric Operating Modes and Sensitivity

Possible operating modes are pitch–catch (acts either as sensor or actuator) and pulse–echo (acts as both sensor and actuator). An important property of a sensor is the sensitivity g.t, which is the open circuit charge developed due to applied stress, where t is the thickness of the piezoelectric element. The coefficient g is related to the piezoelectric coefficient d by the relation $g=d/\left(\epsilon_{r}\epsilon_{0}\right)$, where $\epsilon_{r}$ is the relative permittivity of the piezoelectric element and $\epsilon_{0}$ is the permittivity of free space. This relation is a coefficient realized from Equation (3) by ignoring the indices and noting that the external field $E_{n}$ is zero and D=ϵE, where $\epsilon =\epsilon_{r}\epsilon_{0}$ and E is the generated electric field. This sensing condition yields E=gσ, relating applied stress to the electric field generated across the piezoelectric element. In hydrophone applications, properties such as $d_{33}$ and $d_{31}$ are taken into account due to equal stress from all directions in the piezoelectric element by the figure of merit (FOM) relation $d_{h}g_{h}$, where $d_{h}=d_{33}+2d_{31}$ and $g_{h}=g_{33}+2g_{31}$. The piezoelectric charge coefficient $d_{ij}$ characterizes transmission capability, whereas the piezoelectric voltage coefficient $g_{ij}$ implies reception capability. Both coefficients are to be maximized when the same transducer is used for transmission and reception (for example, the pulse–echo measurement technique). In low-intensity ultrasound applications, $g_{33}$ is given more importance. A large dielectric constant ϵ with relative permittivity of $\epsilon_{r}$~100 plays an important role in electric impedance matching between the transducer and the driving/reception electronics by maximizing the voltage coefficient. Dielectric losses can be considered by relation $d_{h}g_{h}/$tanδ. Substantial acoustic energy is dissipated if the dielectric loss factor tanδ is not minimized [53]. Another property related to losses is the mechanical quality factor $Q_{m}$, which is the amount of mechanical loss due to internal friction or acoustic viscosity. It is desirable for the $Q_{m}$ to be minimum, but in some cases where the generation of short acoustic pulse is required, a compromise is selected $Q_{m}$ = 2–10 to avoid ringing [76].

The electromechanical coupling factor k is another important property in piezoelectric materials that indicates the effectiveness of conversion of electrical energy to mechanical energy and vice versa. The following are the common coupling factors of piezoelectric ceramics [77,78] that are defined as

(5) $$k_{15}=\frac{d_{15}}{\sqrt{S_{44}\epsilon_{11}}}$$

(6) $$k_{33}=\frac{d_{33}}{\sqrt{S_{33}\epsilon_{33}}}$$

(7) $$k_{31}=\frac{d_{31}}{\sqrt{S_{11}\epsilon_{33}}}\;,$$

where subscripts 4 and 5 refer to planes normal to the 1 and 2 axes, respectively. For the thickness mode transducer, the thickness coupling coefficient $k_{33}$ should be high with low planar mode coupling $k_{31}$. The electromechanical coupling factors depend on all the properties required for both sensors as well as actuators such as elastic, piezoelectric, and dielectric properties. Therefore, the electromechanical coupling factor is a good measure of transducer properties of a piezoelectric material operating in pulse–echo mode or pitch–catch (both transmit and receive) mode. The lead zirconate titanate (PZT)-4 material is widely used as an ultrasonic transducer material. The properties of commonly used piezoelectric ceramics and polymers are discussed next.

### 1.3. Commonly Used Piezoelectric Materials as an Active Matching Layer

Material properties greatly influence the performance of piezoelectric devices operating especially at high frequencies. The advent of composites and nanocomposites gave tremendous material combinations to design transducers with a good piezoelectric element and its associated matching layer. Sometimes, piezoelectric material properties are altered to match directly with the propagating media. In such cases, the matched piezoelectric materials are called an active matching layer. This eliminates the requirement of a passive matching layer. A slight altering of piezoelectric materials can facilitate the material selection and design of a passive acoustic matching layer. Therefore, piezoelectric material properties have been very crucial in the optimal design of transducers along with their acoustic matching layers, which this paper summarizes in greater detail. Piezoceramic wafers, piezopolymer films, and piezocomposite materials are commonly used as an active element of sensors, actuators, energy harvesters, and transducers. The electric, mechanical, and acoustic properties differ for bulk and thin-film piezoelectric materials. While bulk materials have been standardized where many different manufacturers report similar properties, thin-film materials differ drastically in the preparation procedure, as the control parameters involved in their processing are many. Therefore, they are not standardized, and it is difficult to list the properties for thin-film sensors. The common bulk piezoelectric materials are listed in Table 1. Piezoelectric substances can be broadly classified as natural materials and synthetic materials. Naturally occurring piezoelectric materials are quartz SiO_2_, berlinite, sucrose, Rochelle salt NaKC_4_H_4_O_6_.4H_2_O, topaz, and a tourmaline group of minerals. Synthetic piezoelectric materials are further classified as synthetic crystals, ceramics, and polymers. Synthetic piezoelectric crystals are gallium orthophosphate (GaPO_4_) and langasite (La_3_Ga_5_SiO_14_) [79]. Synthetic piezoelectric ceramics are barium titanate (BaTiO_3_), lead titanate (PbTiO_3_), lead zirconate titanate (Pb[Zr_x_Ti_1−x_]O_3_ 0 ≤ x ≤ 1), potassium niobate (KNbO_3_), lithium niobate (LiNbO_3_), lithium tantalate (LiTaO_3_), sodium tungstate (NaWO_3_), zinc oxide (ZnO), aluminium nitride (AlN), scandium–aluminum nitride [80], etc. Synthetic piezoelectric polymers are materials such as polyvinylidene fluoride (PVDF) and copolymers of vinylidene fluoride (VDF) with trifluoroethylene (TrFE), or with tetrafluroethylene (TeFE) [10,11,81]. The common bulk piezoelectric materials, their processing techniques, and the resulting acoustic and electric properties needed for acoustic impedance matching are explained next.

#### 1.3.1. Piezoelectric Ceramics

The bulk piezoceramic is prepared by high-temperature sintering at 600–700 °C [82] yielding high density and non-piezoelectric ceramics that constitute ferroelectric crystallites with random orientation. These non-piezoelectric ceramics are made piezoelectric by poling treatment in a chosen direction to align the electric dipoles. Poling is done under a realizable high electric field typically below the breakdown field 2–5 kV/mm and below the ferroelectric Curie point at 80–150 °C [83]. This poling direction governs the vibration modes shown in Figure 3. To influence the domain structure for the enhancement of piezoelectricity, the poled ceramics are cooled through the Curie temperature. The electroding is done by depositing metals such as gold, silver, chromium, or nickel.

Out of the available piezoceramic materials, lead zirconate titanate PZT (Pb[Zr_x_Ti_1−x_]O_3_) (PZT) founded by Jaffe et al. [84] is most widely used because of its strong and stable piezoelectric characteristics, high strength, high Curie point (temperature above which they are non-polar), and ease of fabrication [77]. Most piezoelectric ceramics are based on nominal composition 52–54 mol% lead zirconate and 46–48 mol% lead titanate, which is called PZT. This composition is doped with different elements by different manufacturers with proprietary formulas for different compositions. Some commonly used compositions are PZT-5 with Nb-doping, PZT-6 with Cr-doping, PZT-7 with La-doping, and PZT-4 with Fe-doping. The properties of these piezoelectric ceramics commonly employed by transducer designers can be found in Ref [77,85] and have been listed in Table 1.

Most PZT ceramics are characterized by high elastic moduli, high dielectric constant, low elastic and dielectric loss and high electromechanical coupling factor. However, during processing stages such as calcination and sintering, lead oxide vaporizes in environment accumulating in organisms and causing damage to the nervous system. Also, these are potentially toxic during manufacturing, use and mainly disposal [86]. Thus, lead-free piezoelectric materials such as Barium Titanate (BaTiO_3_), Lithium Niobate (LiNbO_3_)**,** Sodium Bismuth Titanate (Na_0.5_Bi_0.5_TiO_3_), Potassium Bismuth Titanate (K_0.5_Bi_0.5_TiO_3_), Potassium Sodium Diniobate (K_0.5_Na_0.5_NbO_6_), Bismuth Ferrite (BiFeO_3_) whose properties are comparable to that of PZT have attracted lots of attention [87,88,89]. The overall piezoelectric properties of lead-free materials such as barium titanate, lithium niobate and potassium sodium diniobate are slightly lower [90] when compared with PZT materials as seen in Table 1. The natural materials such as quartz and Rochelle salt have very lower piezoelectric properties when compared to synthetic materials [85]. Therefore, synthetic materials are preferred for transducer applications unless other acoustic properties outweigh the electric properties for some applications.

Due to the inherent brittle nature of piezoelectric ceramics and piezoelectric crystals, piezoceramic thin films [91,92] and coatings [93,94] are being explored. High-frequency imaging (>20 MHz), micro-sensors or actuators, and energy harvesting require thick and thin piezoceramic films. The scanning electron microscope uses ultra-thin piezoceramic films with the thickness of around 6–20 μm. Piezoceramic thin films are difficult to fabricate and have inferior piezoelectric properties. Piezoelectric film fabrication techniques such as screen-printing, tape-casting, aerosol deposition, composite sol–gel, electrophoretic deposition, and ink-jet printing produce piezoelectric films with thickness <50 µm preserving the piezoelectric properties of bulk materials. The Lead Magnesium Niobate-Lead Titanate-Lead Zirconate Titanate (PMN–PT–PZT) composite films have been developed using the sol–gel fabrication technique for 200 MHz transducer applications [95]. A range of dielectric constants 1000–5000 can be manufactured using the PZN-PT system that enables optimum electrical impedance matching by tuning it [78]. Ultra-high $d_{33}$ = 2000 pC/N associated with the PZN–PT system suits their use in high energy density and a high-frequency actuator. The PZN–PT piezoelectric materials are single crystals whose machining is difficult.

#### 1.3.2. Piezoelectric Polymers

The low yield strains, high brittleness, high density, high acoustic impedance, toxic nature, and high manufacturing cost of piezoelectric ceramics have limited their use in many applications. Following the discovery of piezoelectricity in stretched and poled PVDF films by Kawai in 1969 [96], piezopolymers have found space in such applications especially related to sensing. Later, piezoelectricity was also found in polymers, such as polyurethane [97]. Single crystalline films of ferroelectric copolymers of vinylidene fluoride and trifluoroethylene P(VDF/TrFE) were prepared by Ohigashi et al. [98]. The studies have been extended to polymers and copolymers of vinylidene cyanide, vinylacetate, polyvinylidene cyanide copolymers, aromatic and aliphatic polyureas, polyvinyl chloride, aromatic polyamides (odd nylons), PVDF copolymers with trifluoroethylene (P[VDF-TrFE]), tetrafluoroethylene (P[VDF-TFE]), hexafluoropropylene (P[VDF-HFP]), PVDF blends with polymethyl methacrylate (PMMA), polyvinyl fluoride, polyvinyl acetate, and ferroelectric liquid crystal polymers [99]. Advantages of piezoelectric polymers are their low dielectric constant, low acoustic impedance [100], excellent insulation properties, flexibility, low cost, biocompatibility, toughness, chemical inertness, mechanical durability, abrasion resistance, nontoxic nature, and radiation stability. The current manufacturing capabilities and lower piezoelectricity compared to piezoceramics limit the piezoelectric polymers to thin-film applications, restricting their use as sensors. Non-polymer piezoelectric materials have very high acoustic impedance when compared to water and tissue. For water immersion-based NDT and biomedical ultrasound, matching layers are employed to improve the coupling efficiency. However, this introduces insertion loss and attenuation. Taking advantage of good acoustic impedance matching with water and biological tissue, piezopolymers have also been considered as suitable materials for transducer applications.

At present, stretched and poled PVDF has the strongest piezoelectric response of all polymers. The PVDF films are prepared from powder or granules using the solvent casting method [104] or melt crystallization (such as the hot press method [11] and melt extrusion [105]). For MEMS applications, the less expensive lithography technique is available [106]. The PVDF can exist in at least four different phases called α, β, γ, and δ [105,107,108,109,110]. Solvent or melt crystallization below 160 °C results in apolar α-phase PVDF. The stretching of PVDF films 4–5 times at 80 °C transforms the apolar α phase to polar β phase [107,111,112]. The stretched β phase is polar, but the dipoles of the crystals are oriented randomly. The PVDF is poled using thermal or corona poling [113,114] in the desired direction depending on the desired mode of vibration. 

The P(VDF-TrFE) synthesized with different TrFE molar ratios crystallize directly in the ferroelectric phase without stretching [115]. It possesses a higher degree of ferroelectric and piezoelectric properties than PVDF due to the higher degree of crystallinity. However, poling is required to impart piezoelectricity in these films. The properties of piezopolymers depend on phase and poling. In addition, since the Curie point of piezopolymers is lower, the temperature during any further stages of transducer fabrication should be the lowest possible to prevent any loss of piezoelectricity due to depolarization and any change of material phase. The properties of bulk piezopolymers are different than those of thin films [116]. The piezoelectric properties of PVDF and P(VDF-TrFE) materials discussed in [74,113,117,118,119] and [120] respectively are given in Table 2. Piezopolymers have an intrinsically low Q factor requiring very light damping, which is easily achievable with air backing. Such property gives a superior damping factor with wider bandwidth when compared to piezoceramic materials. The piezopolymers outperform the piezoceramics by the sensing performance reflected by the g constants.

#### 1.3.3. Piezoelectric Composites

Piezopolymer films such as PVDF and P(VDF-TrFE) have good flexibility but are poor piezoelectric performers as compared to piezoceramic wafers. The mechanical and piezoelectric properties of piezopolymer films also degrade with thermal exposure [122,123]. For some designs, maximizing the piezoelectric sensitivity and minimizing the density becomes necessary to obtain a good acoustic matching with media such as water. In some designs, the transducer must be made mechanically flexible to conform to a curved surface. In some cases, matching layers alone do not achieve proper impedance matching for which the properties of the piezoelectric element need to be modified. Such modification can reduce the piezoelectric properties slightly but can provide great acoustic impedance matching with the wave propagation media. Thus, in such situations, designers often face difficulty in finding suitable materials, as a single-phase material in nature that simultaneously satisfies these requirements is unavailable.

Piezocomposite transducers have been developed further to overcome several drawbacks of piezoceramics such as the brittleness of piezoelectric ceramic, the low sensitivity of piezopolymers, and acoustic impedance mismatch with wave propagating media. For hydrophone applications, piezocomposites offer the required anisotropy to sense the varying hydrostatic pressure, which is otherwise not possible by piezoceramics. This is because for most of the piezoceramics, $d_{31}=d_{32}$ and $d_{33}\approx -2d_{31}$, for which $d_{h}\approx 0$ [124]. Piezocomposites can be tailored for low acoustic impedance, fewer spurious modes [125], and an intermediate dielectric constant.

**Figure 4:**
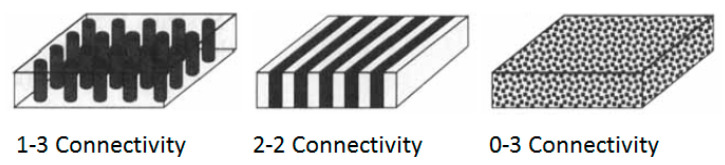
Common piezocomposite configurations used in transducers and their connectivity [103]. Reprinted by permission of the publisher (Taylor & Francis Ltd, http://www.tandfonline.com ).

Most flexible piezocomposites have two phases, a stiff phase comprising piezoceramic or piezo crystal and a soft phase comprising the polymer. A piezocomposite is a diced ceramic with polymer-filled spaces [126]. The composite has the flexibility to provide heat dissipation or structural support. The material has natural damping and can be easily designed as an array. The characteristic acoustic impedance is around 10 MRayl, which is much closer to water and tissue. There are 10 original notations and 16 extended notations [127] to describe the composites, depending on the connectivity of a number of dimensions associated with each phase [128,129,130]. Some of them such as 0-0, 1-0, 2-0, 3-0, 1-1, 2-1, 3-1, 2-2, 3-2, or 3-3 are internationally accepted (see Figure 4). The first digit refers to the number of dimensions of connectivity for the piezoelectric phase, and the second digit refers to the electromechanically inactive polymer phase. 

An array of piezocomposite transducers has been made by blending piezo powder, piezo-rods, or piezo fibers with various resins to simultaneously impart flexibility and higher sensitivity. To achieve higher piezoelectric properties, piezopolymers are used as active resin (or matrix) [121]. Recently, 1-3, 0-3, and 2-2 piezocomposites are commonly explored due to their importance in military and commercial applications [131]. Methods of fabricating 1-3 piezocomposites such as dice-and-fill, injection molding, lost mold, and others are relatively complex [132,133]. With the computer-aided design flexibility and additive manufacturing technology, this problem is being solved [133]. The preparation of 0-3 composites is relatively simple and involves mixing inorganic particles in polymer followed by curing [134]. 

Poled film sheets made of PZT powder and epoxy resin mixture show superior performance as compared to the PVDF sensors of the same dimensions [135]. Egusa and Iwasawa [136] developed piezoelectric paint using a PZT powder as filler and epoxy resin as a binder. They tested its ability as a vibration sensor at frequencies up to 1 MHz. Active Fiber Composite (AFC) transducers have extruded piezoceramic fibers embedded in an epoxy matrix and have interdigitated electrodes that are symmetrically arranged on both surfaces [137]. Due to the presence of fine ceramic fibers and increased specific strength, conformability to curved surfaces is observed in AFC as compared to monolithic piezoceramic materials. Similar to AFCs, macro fiber composites (MFCs) have fibers but have a rectangular cross-section instead of a circular shape to significantly reduce the small-batch manufacturing costs [138]. The transverse elastic modulus $E_{t}$ can be estimated by the Reuss model [139] for piezocomposites utilizing piezoelectric rods or fibers given by

(8) $$E_{t}=\frac{V_{f}}{E_{f}}+\frac{V_{m}}{E_{m}}\;,$$

where $V_{f}$ and $V_{m}$ are the volume fraction of the piezoelectric fibers and matrix, respectively. $E_{f}$ and $E_{m}$ are the Young’s modulus of the fiber and matrix, respectively. The longitudinal elastic modulus $E_{l}$ along the direction of the rod or fiber can be estimated by the Voigt constant strain model [139] given by

(9) $$E_{l}=V_{f}E_{f}+V_{m}E_{m}.$$

A detailed discussion on the estimation of coupling coefficient and quality factor for piezocomposite-based transducers is discussed in [140,141], especially for hydrostatic transducers. Although analytical and numerical models are available to estimate the material properties of piezocomposites [142,143,144,145], they are ineffective due to the complexity of their processing parameters and resulting variations in the material properties [146]. For this reason, it is not possible to find all measured values of electric and mechanical properties in the literature. This problem leads to the designer’s inability to compare the performance of different materials for transducer design via acoustic and electric impedance matching. Some common piezocomposites used for transducer applications have been listed in Table 3 with their piezoelectric and acoustic properties. Table 4 lists some other common transducer materials. The highest coupling coefficients of piezocomposites favor their use in energy-harvesting applications. The MFCs outweigh other types of piezocomposites in most aspects including the hydrostatic sensing parameter $d_{h}$. For this reason, they are the most explored materials at present.

Piezocomposites as transducers have gained high importance since the early 1980s [140,147] as they do not require a matching layer and pose less dependence on the matching layer properties. Composite piezoelectric materials with several connectivity patterns provide improvement in the desired properties of piezoelectric element for applications involving acoustic matching with hydrostatic conditions. The major reason is due to the capability of tailoring the density and directional elastic properties. Such acoustic matching results in low Q (3–10) with high bandwidth and better pulse reproduction. The acoustic properties of composites developed specifically for acoustic impedance matching will be discussed in Section 3 in greater detail.

### 1.4. Theory of Acoustic Impedance Mismatch and Transducer Performance Parameters

**Figure 5:**
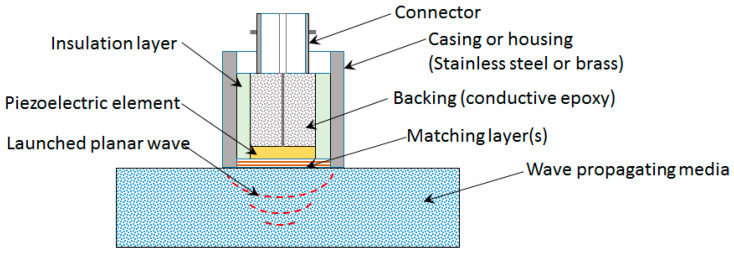
Schematic of a piezoelectric ultrasonic transducer cross-section showing various parts.

Consider a typical bulk wave ultrasonic transducer shown in Figure 5 with its main parts such as the piezoelectric element, backing material, matching layers, casing, and electrical connector with contacts. Some transducers may have an electrical matching and tuning network housed inside their casing. One or more acoustic impedance matching layers at the front face of the transducer serve as the wear plates. The transmission media can be the intended wave receiving object or intermediate couplants such as gels, air, and water. The thickness and the acoustic impedance of the piezoelectric element, backing material, and matching layers govern the center frequency and bandwidth of such a transducer.

The huge impedance mismatch between the piezoelectric element made of ceramic and the surrounding wave propagating media (tissue, air, water, etc.) results in low sensitivity and a narrow bandwidth, significantly lengthens ultrasound pulses, and lengthens transducer ring-down time [51,163,164,165]. This effect is also similar to the unmatched backing layer. Table 4 shows the relative difference of acoustic impedance between the piezoelectric element and the propagation media. The reflection ${\dot{u}}_{R}$ and transmission ${\dot{u}}_{T}$ wave velocity for normal incidence is given by [71]

(10) $${\dot{u}}_{T}=\frac{2Z_{A}}{Z_{A}+Z_{B}}{\dot{u}}_{I}=R_{T}{\dot{u}}_{I}$$

(11) $${\dot{u}}_{R}=\frac{Z_{B}-Z_{A}}{Z_{B}+Z_{A}}{\dot{u}}_{I}=R_{R}{\dot{u}}_{I}\;,\;$$

where $u_{I}$ is the incident wave velocity, $R_{T}$ is the ratio of transmitted wave amplitude, and $R_{R}$ is the ratio of reflected wave amplitude. The acoustic impedance Z is the product of acoustic velocity v and density ρ of the wave propagating media. It is given by [166]

(12) Z=vρ.

The bulk wave velocity v, also called longitudinal wave velocity in isotropic solids, is related to Young’s modulus E and Poisson’s ratio ϑ as [156]

(13) $$v^{2}=\frac{E\left(1-\vartheta \right)}{\rho \left(1+\vartheta \right)\left(1-2\vartheta \right)}=\frac{K+4G/3}{\rho }=\frac{\chi +2\mu }{\rho }\;,\;$$

where K is the bulk modulus, G is the shear modulus, and χ and μ are Lame’s constant. Acoustic impedance is directionally dependent, especially in composites, as the acoustic velocity differs along with different directions. Acoustic impedance is also expressed in terms of basic material properties as the ratio of acoustic pressure P to the product of velocity v and surface area A as

(14) $$Z=\frac{P}{vA}.$$

Specific acoustic impedance is the most important property in tissue imaging, and it is defined as the product of acoustic impedance and surface area ZA=P/v. Acoustic impedance for the specific area $Z_{0}$ in terms of the open circuit elastic constant $C_{33}^{D}$ for a thickness mode transducer is given by [155]

(15) $$Z_{0}=\rho Av=A\sqrt{\rho C_{33}^{D}}.$$

The acoustic impedance can be expressed in terms of elastic constants as [167] $E=Z^{2}\left(1+\vartheta \right)\left(1-2\vartheta \right)/\left[\rho \left(1-\vartheta \right)\right]$. The acoustic impedance of materials governs the energy transfer from the piezoelectric element to wave propagation media and vice versa. Often large differences cause a reflection of acoustic energy resulting in low energy transmission by waves. The amplitude reflection coefficient R and transmission coefficient T depend on the acoustic impedance of materials in medium 1 from which the wave originates and transmits to medium 2 as given by [162]

(16) $$T=\frac{A_{T}}{A_{I}}=1+\frac{Z_{2}-Z_{1}}{Z_{2}+Z_{1}}$$

(17) $$R=\frac{A_{R}}{A_{I}}=\frac{Z_{2}-Z_{1}}{Z_{2}+Z_{1}}\;,$$

where $Z_{1}$ and $Z_{2}$ are the acoustic impedances of medium 1 and 2 respectively. $A_{T}$, $\;A_{R}$, and $A_{I}$ are the amplitudes of transmitted, reflected, and incident waves, respectively. The reflected waves produce oscillations in the transducer if damping is absent. Damping is usually achieved by the backing layer. If $A_{1}$ and $A_{2}$ are amplitudes of the first and second cycle of oscillation, then the damping coefficient is given by

(18) $$\eta =\frac{A_{1}}{A_{2}}=\{\begin{array}{cc} \frac{\left(Z_{p}+Z_{b}\right)\left(Z_{p}+Z_{m}\right)}{\left(Z_{p}-Z_{b}\right)\left(Z_{p}-Z_{m}\right)},\;\; & Z_{p}>Z_{b},Z_{m}\;\text{or}\;Z_{p}<Z_{b},Z_{m},\\ \frac{{\left(Z_{p}+Z_{b}\right)}^{2}{\left(Z_{p}+Z_{m}\right)}^{2}}{{\left(Z_{p}-Z_{b}\right)}^{2}{\left(Z_{p}-Z_{m}\right)}^{2}},\;\; & Z_{b}>Z_{p}>Z_{m}\;\text{or}\;Z_{b}<Z_{p}<Z_{m}, \end{array}$$

where $Z_{p}$, $Z_{b}$, and $Z_{m}$ are the acoustic impedances of the piezoelectric element, backing layer, and matching layer, respectively. The damping coefficient is related to the mechanical quality factor Q by

(19) $$Q=\frac{\pi }{ln\;\eta }.$$

A −3 dB bandwidth BW is easily estimated by modeling the transducer as a mass suspended by spring in a damped media. The relation for BW discussed by Krautkramer and Krautkramer [168] is given by

(20) $$BW=\frac{f_{R}}{Q}\;,$$

where $f_{R}$ is the resonant frequency of the transducer. The bandwidth can be increased by matching the characteristic impedances of the backing and front-end layers with the piezoelectric element. Acoustic matching with the backing material increases η and therefore increases the energy loss. Thus, BW is enhanced at the expense of transducer efficiency. If η is not large, $f_{R}$ is similar to the characteristic frequency of the transducer $f_{0}$ given by

(21) $$f_{0}=\frac{v}{2d}$$

where d is the thickness of the transducer. The BW can also be estimated from the experimental frequency response obtained using standard test equipment given by [169]

(22) $$BW=\frac{f_{u}-f_{l}}{f_{c}}$$

where $f_{l}$ and $f_{u}$ are the lower and upper −6 dB frequencies. The center frequency of the Fast Fourier Transforms (FFT) spectrum $f_{c}$ is given by

(23) $$f_{c}=\frac{f_{u}+f_{l}}{2}.$$

Similarly, the effective electromechanical coupling factor can be expressed in terms of resonance $f_{r}^{2}$ and anti-resonance $f_{a}^{2}$ frequencies measured from experiments as

(24) $$k_{eff}=\sqrt{\frac{f_{a}^{2}-f_{r}^{2}}{f_{a}^{2}}}.$$

As per the IEEE standard, the thickness mode electromechanical coupling coefficient ($k_{t}$) is given by [170,171,172]

(25) $$k_{eff}=\sqrt{\frac{\pi f_{r}}{2f_{a}}\tan\left(\frac{\pi }{2}\frac{f_{a}-f_{r}}{f_{a}}\right)}.$$

Insertion loss for a transducer operated in a pulse–echo mode (using the same electronics for actuation and reception) can be measured by [36]

(26) $$IL=10\log\left(\frac{P_{o}}{P_{i}}\right)=10\log\left(\frac{V_{0}^{2}/R_{o}}{V_{i}^{2}/R_{i}}\right)=20\log\left(\frac{V_{o}}{V_{i}}\right),$$

where $V_{o}$ and $V_{i}$ are the echo voltage and excitation voltage, respectively. $P_{o}$ and $P_{i}$ are the transducer output power and transducer input power, respectively. Impedance matching layers mitigate the enormous impedance mismatch between the piezoelectric element and the transmission media. The reduction or elimination of acoustic mismatch results in the efficient energy transfer greatly increasing the performance of ultrasonic sensors and transducers. Several design procedures for acoustic matching layers will be discussed in Section 2. Many equations discussed in this section are not directly related to the acoustic matching applications but are required to estimate properties that are useful to design acoustic impedance matching layers and serves as a guideline or data for the design process. These also serve to better understand the acoustic parameters and their relations. As the review paper is specifically concerned with acoustic matching for acoustic applications, the general acoustic applications have been briefly discussed in this section. Acoustic matching is not carried out commonly due to the difficulty associated with finding the required guidelines. Therefore, the applications involving acoustic matching are limited, and these limited applications have been covered wherever possible. Section 1 mainly provides guidelines for the designer thereby filling the gap, which prevents the design of acoustic impedance matching for the rapidly evolving broad range of applications such as the internet of things. Special acoustic matching techniques for specific acoustic applications have been covered exclusively in Section 3 and Section 4.

## 2. Acoustic Matching Tools and Methods

Although acoustic matching layer design has been studied extensively with the proposal of multiple matching layers with optimum properties, such materials can be difficult to obtain. In addition, the requirement of very thin layers at high frequencies increases the manufacturing cost considerably or makes the design process difficult. In addition, achieving high sensitivity with broad bandwidth remains a challenge [52,173]. Last but not the least, attenuation increases with the addition of the matching layers. The matching configurations available for bulk wave transducers are half-wavelength (λ/2) [174], single quarter-wavelength (λ/4), one-eighths λ/8 [175], and similar configurations to λ/4 such as (*n* + 1)λ/4 [176], stacks of λ/4 layers [177], and a stack of very thin matching layers whose total acoustic thickness is λ/4. Quarter wavelength matching is a traditional mechanism requiring a specific acoustic impedance and thickness equivalent to quarter wavelength [178,179,180]. The outer layer receives key attention during the design of matching involving multiple layers. The outer layer needs to closely match the acoustic impedance of the surrounding media. The availability of consistent materials with very low acoustic impedance, very low attenuation, and the desired thickness for the designed geometry and frequency is limited. Most matching techniques involve the design of layers for single operating frequency. The acoustic matching techniques can be mainly classified as traditional method, transmission line method, and discrete element-based spring mass technique. While traditional methods are simple and straightforward, transmission line theory is more advanced and accurate. Certain parameters are difficult to determine using transmission line theory for which the spring mass technique is introduced, which uses transmission line theory. These acoustic matching methods are discussed below.

### 2.1. Traditional Quarter Wavelength Matching Method

Acoustic impedance matching using quarter-wavelength λ/4 matching layers without extensive dependence on any specific material impedance can be done using two ways with slightly different results [176]. The first way is a traditional way involving the optimization of energy transmission at two interfaces involving different media with an intermediate matching layer [181]. This method relies on the specific impedance of the matching layer material. For example, the piezoelectric element, matching layer, and wave propagation media having acoustic impedances $Z_{A}$, $Z_{M}$, and $Z_{B}$ respectively. The piezoelectric element acting as a transmitter and/or receiver is shown in Figure 1. The transmitted wave to the wave propagation media through the matching layer is the sum of contributions due to each multiple reverberation. A quarter-wavelength matching layer ensures that all transmitting reverberations have the same phase, thereby ensuring constructive and destructive interference [176,182,183,184]. The ratio of transmitted energy flux to the incident energy flux is given by

(27) $$\Upsilon_{T}={\left(\frac{T_{A}T_{B}}{1-R_{A}R_{B}}\right)}^{2}\frac{Z_{B}}{Z_{A}}\;,\;$$

where,$\;T_{A}=2Z_{A}/\left(Z_{A}+Z_{M}\right)$ and $T_{B}=2Z_{M}/\left(Z_{M}+Z_{B}\right)$ are the transmitted wave ratios, and $R_{A}=\left(Z_{M}+Z_{A}\right)/\left(Z_{M}+Z_{A}\right)$ and $R_{B}=\left(Z_{B}+Z_{M}\right)/\left(Z_{B}+Z_{M}\right)$ are the reflected wave ratios. The energy transmission through the matching layer is at its maximum if the matching layer has an acoustic impedance $Z_{M}$, which is the geometric mean of the acoustic impedance [155,185] of the two media given by

(28) $$Z_{M}=\sqrt{Z_{A}Z_{B}}.$$

Multiple matching layers can be employed to maximize the transmitted energy when attenuation due to reverberation losses are low within the matching layers. This results in an improved spectral performance of the transducer [159,186,187,188]. Quarter-wavelength matching involving n matching layers has an acoustic impedance in any $j^{th}$ layer given by [176]

(29) $$Z_{M}^{\left(j\right)}=\sqrt[{n+1}]{Z_{A}^{\left(n-j+1\right)}Z_{B}^{\left(j\right)}}.$$

Oakley [189] has considered the effect of noise due to the thermal effect and amplifier to design a piezoelectric element and a matching layer for the transducer using the Krimholtz, Leedom, and Matthaei (KLM) model.

### 2.2. Transmission-Line Approach

Another way of determining the quarter-wavelength matching layer properties is by imposing the optimum bandwidth and maximum efficiency using of the Krimholtz, Leedom, and Matthaei (KLM) transmission line model [71]. The effect of backing and matching layers together with insertion loss in essential to obtain good impulse response [182]. This ensures high sensitivity, broad bandwidth with low ripple, and short duration. A common case of high-frequency matching involves the matching of high-performance piezoelectric materials (lithium niobate (LiNbO_3_), lead zirconate titanate (PZT), and lead magnesium niobate-lead titanate (PMN–PT)) with medium such as water or tissue [71]. In such a case, tuning the acoustic properties of the matching layer material involves mixing a high-impedance material with a low-impedance polymer with a certain ratio. The fabrication accuracy is high at low frequency, as the wavelength is large. At a high frequency, the quarter-wavelength requirement of the matching layer becomes impractical to achieve. The reasons being (1) a noticeable variation of thickness compared to a very small wavelength, (2) surface roughness, and (3) a variation of material properties within the layer as the particle size approaches the wavelength. Therefore, a method based on microwave transmission line theory is developed to design the acoustic matching layers as an electric impedance matching network [190]. At high frequencies and for broadband impulse actuation, the piezoelectric material must be considered as a transmission line to design the matching layers. Otherwise, considerable acoustic attenuation needs to be introduced to obtain a good impulse response by minimizing the ringing effect, which results in a Gaussian shape response [191]. The KLM method-based transmission line approach takes account of such considerations. The microwave transmission line method uses the KLM method to design the matching network, which is discussed first.

**Figure 6:**
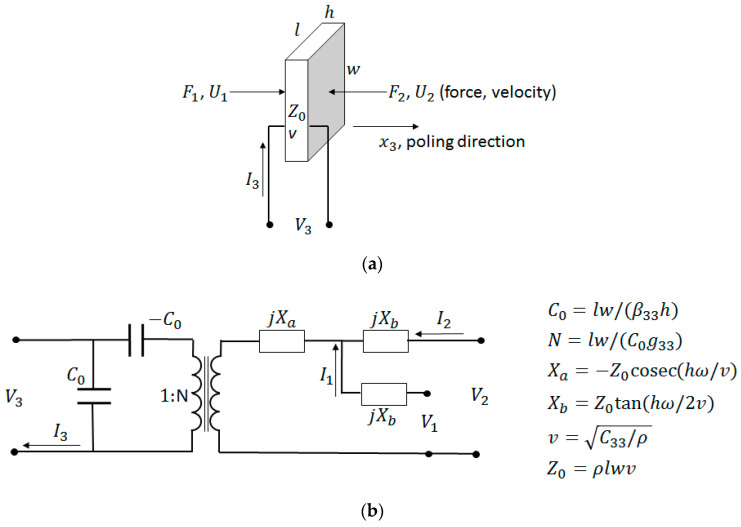

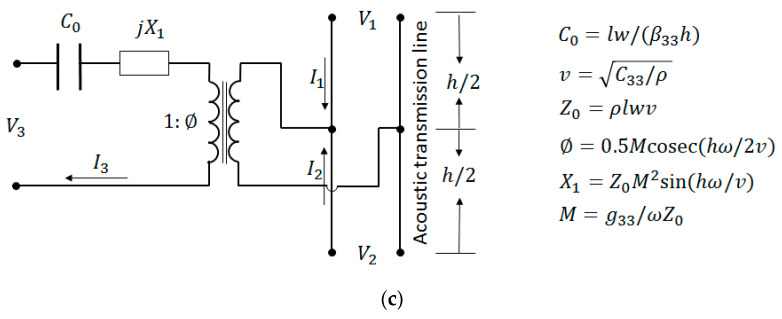
(**a**) Schematic of a piezoelectric wafer operating in the thickness mode with its geometry showing the poling direction, voltage across electrodes $V_{3}$ and direction of forcing ($F_{1},F_{2}$ ), and velocity ($U_{1},U_{2}$ ). (**b**) Mason’s electric equivalent circuit. (**c**) Krimholtz, Leedom, and Matthaei (KLM) electric equivalent circuit. Here, ρ = density, ω = angular frequency, β = dielectric impermeability, $C_{33}$ = elastic stiffness, $g_{33}$ = piezoelectric constant, and v = acoustic wave velocity in the direction of acoustic wave propagation.

Consider a piezoelectric wafer vibrating in the thickness mode due to the applied voltage $V_{3}$, as illustrated in Figure 6a. For such a configuration, the Masons model considers the electric effects due to the insertion loss, acoustic load, and backing impedance on the acoustic port (see Figure 6b). However, this method does not consider the effect of piezoelectric wafers cascaded with matching and backing materials. The three elements across the secondary of the transformer are interpreted as an acoustic transmission line resulting from cascading. At the acoustic port, the voltage 1 *V* represents a unit force and current 1 *A* represents a unit velocity. In the Mason’s model, the current is developed across both the transmission line and the secondary of the transformer. This leads to the difficulty in determining the lumped components of the cascaded piezoelectric wafer. Therefore, methods that also include the effect of the matching layer on the acoustic ports using the Masons model [192] are trial-and-error based [182,186,193,194]. The difficulty associated with the Masons model in interpreting the combined effect due to electric and acoustic matching schemes has led to the design procedures based on the KLM model [195,196]. Its electric representation as shown in Figure 6c has a single coupling point through a coupling transformer instead of a cumbersome distributed coupling of the piezoelectric transmission line. Lumped electrical elements and acoustic wave properties can be clearly distinguished for design purposes allowing the connection of an acoustic transmission line of arbitrary impedance connected to the electric port. This allows an easy determination of matching the layers’ properties, since acoustic matching uses transmission line formalism, whereas electrical matching uses lumped components. The effects of the matching layers and their bonding on inductances, capacitances, and electrical resistance can be easily determined and accounted in the design of the transducer.

#### 2.2.1. Single and Multi-Layer Matching

The KLM transmission line model is used to impose optimum bandwidth and maximum efficiency to determine the number of matching layers and their acoustic impedances. The number of matching layers is first determined using the acoustic impedances of the piezoelectric element $Z_{0}$ and propagating media $Z_{B}$ and the piezoelectric coupling coefficient of the piezoelectric element $k_{t}$. For a single quarter-wavelength matching layer (λ/4), the acoustic impedance of the matching layer $Z_{M}$ as proposed and modified for broadband considerations by Desilets et al. [71,197] is given by

(30) $$Z_{M}=Z_{0}^{1/3}Z_{B}^{2/3}.$$

Similarly, for double λ/4 layers, the acoustic impedance of the first matching layer $Z_{M1}$ is given by [157]

(31) $$Z_{M1}=Z_{0}^{4/7}Z_{B}^{3/7}$$

and the acoustic impedance of the second matching layer $Z_{M2}$ is given by

(32) $$Z_{M2}=Z_{0}^{1/7}Z_{B}^{6/7}.$$

For a single layer, matching the energy transfer from the matching layer to the propagating media based on the transmission line theory is given by [167]

(33) $$T_{E}=1-{\left(\frac{Z_{B}-Z_{M}}{Z_{B}+Z_{M}}\right)}^{2}.$$

#### 2.2.2. Cascaded Layer Matching

**Figure 7:**
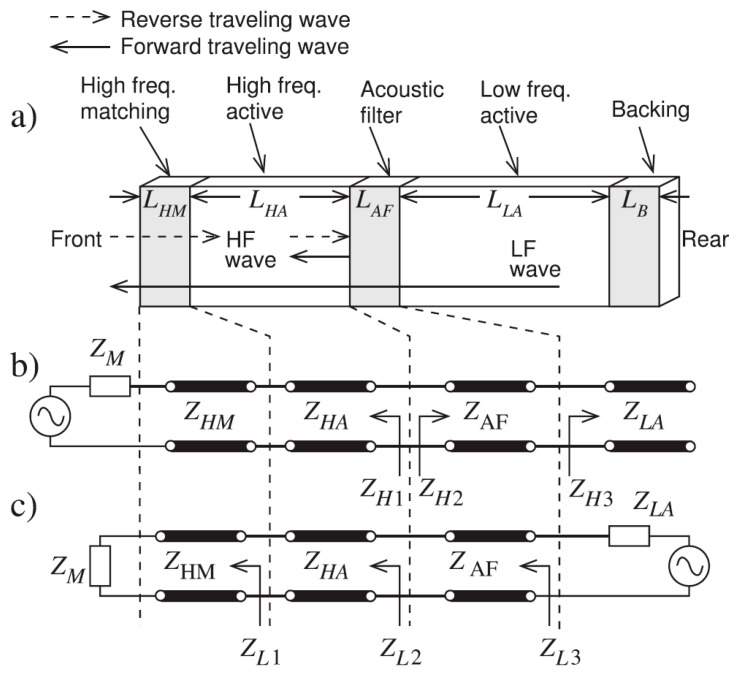
Schematic of piezoelectric cascaded structure and its equivalent circuit with the ability of (**a**) dual-frequency filter design, (**b**) high-frequency reception, and (**c**) low-frequency transmission. Reprinted from [190], with the permission of AIP Publishing.

Using the KLM model-based electric representation of cascaded matching layers, the matching layers are designed based on quarter-wavelength criteria for the efficient transmission of wave components of selected frequency. This idea is based on the acoustic filter reported by Ma et al. [190], which had dual-frequency filtering capability with high-frequency reception and low-frequency transmission. The acoustic filter design uses microwave transmission-line theory [198,199,200], which transmits low-frequency waves and receives high-frequency waves, thereby blocking the reception of low-frequency waves completely. The schematic of such a dual-frequency acoustic filter used in super-harmonic microscopy is shown in Figure 7a. Thus, for low-frequency and narrow-banded excitation, the wave components transmit very efficiently with minimal reflections. The high-frequency (HF) band-stop filter is designed by creating mismatch by introducing $Z_{H1}$ and $Z_{H2}$, which is practically fulfilled by ensuring the HF wave pulse length to be shorter than twice the length of the *LF* element thickness (See Figure 7b). Assuming matched backing $Z_{H3}=Z_{LA}$, the input impedance at the front end of AF layer is given by

(34) $$Z_{H2}=Z_{AF}\frac{Z_{LA}\cosh\left(\gamma_{HF}l\right)+Z_{AF}\sinh\left(\gamma_{HF}l\right)}{Z_{AF}\cosh\left(\gamma_{HF}l\right)+Z_{LA}\sinh\left(\gamma_{HF}l\right)},$$

where $\gamma_{HF}=\omega /v_{s}$ is a propagation constant, l is the thickness of the layer, and $\gamma_{HF}=\alpha_{HF}+i\beta_{HF}$. When the matching layer is thin with low attenuation, the attenuation coefficient $\alpha_{HF}$ = 0. The transmission coefficient of intensity through the matching layer *AF* is given by

(35) $$T_{I}=\frac{4Z_{HA}Z_{LA}}{{\left(Z_{HA}+Z_{LA}\right)}^{2}\cos^{2}\left(\beta_{HF}l\right)+{\left(Z_{AF}+Z_{HA}Z_{LA}/Z_{AF}\right)}^{2}\sin^{2}\left(\beta_{HF}l\right)}\;,$$

when $\beta_{HF}l=\left(2n+1\right)\lambda_{HF}/4$ with n=0,1,2,…., the matching layer *AF* functions as a quarter wavelength impedance transformer as Equation (34) reduces to $Z_{AF}=\sqrt{Z_{H2}Z_{LA}}$. Similarly, matching layer *HM* is designed as $L_{HM}=\lambda_{HF}/4$.

The band-pass filter acts as a passive amplifier for the low-frequency acoustic wave, as shown in Figure 7c. The active element *LF* is a voltage stress source with Thevenin’s impedance of $Z_{LA}$. For the low-frequency wave, the medium is considered large with no phase delay between stress and strain for which it can be assumed as a pure resistive load $Z_{M}$. The cascaded element and matching layers are comparable to the wavelength, with each element acting as a part of the transmission line. The input impedance can be calculated using ABCD parameters as

(36) $$\left\{\begin{array}{c} P_{L3}\\ V_{L3} \end{array}\right\}={\left[\begin{array}{cc} A & B\\ C & D \end{array}\right]}_{AF}{\left[\begin{array}{cc} A & B\\ C & D \end{array}\right]}_{AF}{\left[\begin{array}{cc} A & B\\ C & D \end{array}\right]}_{AF}\left\{\begin{array}{c} P_{M}\\ V_{M} \end{array}\right\},$$

where P corresponds to the pressure and V corresponds to the velocity in respective materials. The ABCD matrices with subscripts denote the transmission line section of each layer defined as

(37) $$\left[\begin{array}{cc} A & B\\ C & D \end{array}\right]=\left[\begin{array}{cc} \cosh\left(\gamma_{LF}l\right) & Z_{0}\sinh\left(\gamma_{LF}l\right)\\ \sinh\left(\gamma_{LF}l\right)/Z_{0} & \cosh\left(\gamma_{LF}l\right) \end{array}\right],$$

where $Z_{0}$ is the characteristic impedance of the respective transmission line. In the absence of attenuation, the pressure transmission coefficient is

(38) $$T_{P}=\frac{2Z_{L3}}{Z_{L3}+Z_{LA}^{*}}.$$

In the presence of attenuation, the pressure transmission coefficient is estimated as the scattering parameter $S_{21}$ of a two-port transmission line. Tools such as AWR Microwave Office [201] from AWR corp., EI Segundo, CA, USA and PIEZOCAD [202] from Sonic Concepts Inc., Bothell, WA, USA are available to simulate such circuits for the fine-tuning of a matching network. While the transmission line method allows distributed calculation, an arbitrary number of layers, arbitrary impedances, and a clear display of matching in the Smith Chart, the electromechanical correlation is difficult in some cases. Myhre et al. [203] used a similar cascaded layer concept with matching layers to design acoustic impedance matching layers for a dual-frequency transducer used for imaging and ultrasound therapy.

### 2.3. Mass-Spring Approach

**Figure 8:**
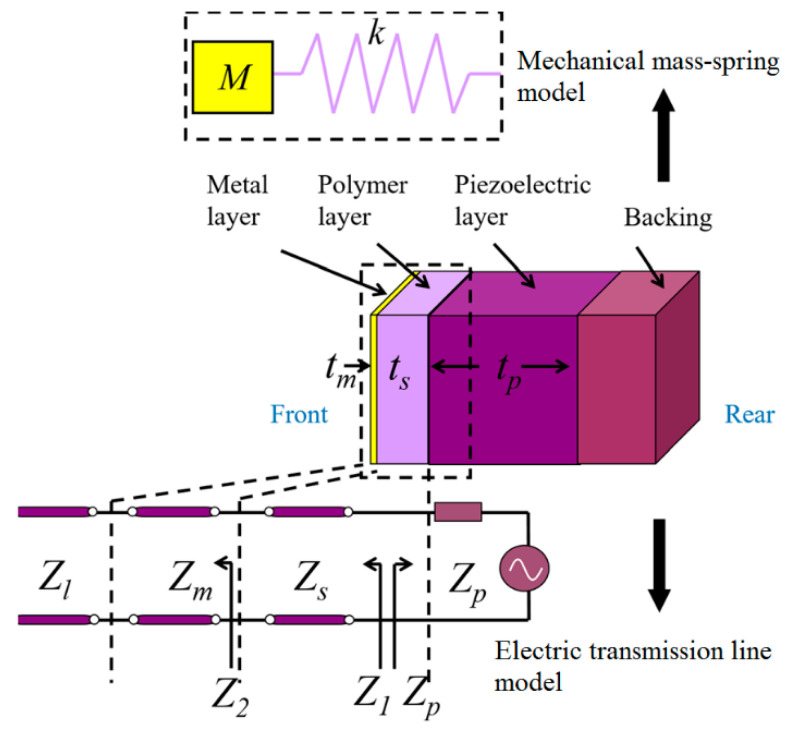
Schematic of a piezoelectric element with two-layer matching showing mechanical (spring-mass) and electrical (transmission line) equivalent models. Reprinted from [154], with the permission of AIP Publishing.

The mass-spring based approach is based on using high impedance material as a mass and low impedance material as spring [204,205,206]. Figure 8 shows the schematic of the two-layer matching provided to a piezoelectric element. Its equivalent mechanical and electric representations as a mass spring and microwave transmission line are shown, neglecting the presence of the damping layer. The equivalent impedance of mass-spring systems is tuned to achieve a resonator system with efficient acoustic wave propagation. The resonant frequency $\omega_{0}$ and acoustic impedance at the driven end of the spring derived by Toda and Thompson [204] for such spring-mass approximation is given by

(39) $$\omega_{0}=\sqrt{K/M}$$

(40) $$Z_{1}=\frac{KM}{Z_{l}}$$

where $Z_{l}$ is the load impedance of the medium such as water, tissue, etc., in which the wave propagates, stiffness $K=\rho_{s}v_{s}^{2}/l_{s}$, and mass with partial influence on wave motion $M=\rho_{m}l_{m}+0.4\rho_{s}l_{s}$. The subscripts s, m, p, and l in various parameters stand for spring, mass, piezoelectric element, and media (as load). For the given piezoelectric spring and mass materials, the thickness of the matching layer constituting of spring and mass can be estimated numerically to resonate at the center frequency of excitation. For a comparison of the spring-mass model with the microwave transmission line model, consider the equivalent electrical impedance seen at each layer as shown in Figure 8. The impedances are given by

(41) $$Z_{2}=Z_{m}\frac{Z_{l}+Z_{m}\tan h\left(\gamma_{m}t_{m}\right)}{Z_{m}+Z_{l}\tan h\left(\gamma_{m}t_{m}\right)}$$

(42) $$Z_{1}=Z_{s}\frac{Z_{2}+Z_{s}\tan h\left(\gamma_{s}t_{s}\right)}{Z_{s}+Z_{2}\tan h\left(\gamma_{s}t_{s}\right)},\;$$

where $\gamma_{s}$ is the frequency-dependent propagation constant. Similar to the spring-mass model where the desired resonant frequency is obtained by tuning the matching layer thickness, the thickness of the matching layers is numerically estimated to generate matched $Z_{1}$ in the case of the microwave transmission line model. In both models, $Z_{1}$ is frequency dependent. The mass-spring approach provides a straightforward approach to estimate lumped elements and the resulting impedance, but its accuracy depends on the assumption of equivalent mass and the requirement of a significant higher impedance of the mass than the spring. It also excludes the consideration of piezoelectric material properties.

### 2.4. Wave Propagation Model

Wave propagation in piezoelectric material, matching layers, backing, and wave propagation media can be modeled in many ways [207,208]. The mass-spring model is based on thin layer approximation where layer thickness $t_{p}<\lambda /4$ and $\omega <\omega_{0}$. Therefore, a more accurate one-dimensional wave propagation model is suggested to be used in such cases. A layer with impedance $Z_{m}$ loaded with impedance $Z_{1}$ on the outer surface or downstream and impedance $Z_{2}$ seen from the inner surface results in a partially reflected wave from the outer surface causing phase delay due to propagation over a distance d. The impedances are related by the relation [204,209,210]

(43) $$Z_{2}=Z_{m}\frac{Z_{1}+iZ_{m}\tan\left(\frac{\omega t}{v}\right)}{Z_{m}+iZ_{1}\tan\left(\frac{\omega t}{v}\right)}.$$

This is a more generic relation that is frequently used in microwave transmission line design, which has been used for the design of both the acoustic impedance of the matching layer and the backing layer absorber. The above equation is applied to the multi-layer structure where the first $Z_{1}$ is considered as radiation impedance and $Z_{2}$ is estimated considering the material parameters of $Z_{m}$. Next, this $Z_{2}$ is considered as a new $Z_{1}$ for the next layer. This sequential calculation of many layers accounts for any number of reflections using polynomials [211].

### 2.5. Backing Absorber

Traditionally, the backing material used is conductive epoxy such as E-solder [212] with high attenuation (120 dB/mm at 30 MHz) and relatively low acoustic impedance (5.9 MRayl). It provides low insertion loss and well-shaped short pulses suitable for medical imaging if designed together with front face matching layers. Nicolaides et al. [213] studied the effect of backing materials such as polyvinyl chloride (PVC), aluminum, brass, fiberglass, and a combination of PVC and brass on the performance of an underwater transducer with 1–3 piezocomposite material. Fiberglass or a combination of PVC and brass backing plates proved to have the best match as backing layers. Tungsten-loaded epoxy [159,214] has higher acoustic impedance 8–20 MRayl. The variation of acoustic impedance is achieved by varying the content of tungsten. The varying acoustic impedance is useful to tune the backing for broad bandwidth. Besides being lossy, the backing materials should be rigid to provide support to the fragile active elements. Grewe et al. [215] showed an increase in filler particle size or a decrease in volume fraction of filler leading to an increase in composite attenuation for a tungsten/vinyl composite backing-based transducer.

The difficulties associated with obtaining both high sensitivity and large bandwidth have led to multiple backing absorber layers. The design method is similar to multiple front-end acoustic impedance matching layer(s). The absorber layer(s) are simultaneously designed with acoustic impedance matching layer(s). As a perfectly matched backing absorber reduces sensitivity, the absorber must be designed with an appropriate mismatch. A heavy-metal/polymer rubber composite serves as a satisfactory material for the backing but the inconsistent mixing causes variation in the desired acoustic impedance properties that affect performance, especially at high frequency [182,216]. Toda and Thompson considered a multi-layer design for acoustic impedance and backing absorber zones for a transducer. They compared results obtained from the mass-spring approach and wave propagation model. Their design consisting of metal–polymer inner layers and a quarter-wavelength outer polymer layer yielded 50%, −6 dB bandwidth at 3.2 MHz. A polymer–metal backing made of 10 layers of copper tape was used as an absorber for a 715 µm thick PZT active element. The attenuation of this material was measured and found to be 222 dB/cm at 3 MHz.

### 2.6. Degradation and Endurance

Most of the transducers do not suffer from degradation unless exposed to high-temperature levels and radiation. Some applications where transducers are subjected to heat and radiation are industrial, thermal power plants, and nuclear power plants [69]. Plastics and its derivatives such as composites have low material and fabrication costs when compared to metals. The properties of plastics or composites such as impedance and attenuation can be tailored for acoustic matching. The most common failure modes due to radiation and thermal exposure have considerable resemblance failures at high temperature [217]. Metals do not exhibit degradation from gamma radiation, which is the main cause of degradation. The properties of an ultrasonic transducer’s backing layer such as acoustic impedance and attenuation can change considerably with radiation exposure. Several plastics such as Teflon used as matching layers should be avoided [218,219,220,221] for use in a radiative environment. Radiation increases the cross-linking of polymers, reducing flexibility and toughness [222]. Swelling, gas production, residual stress, thermal expansion, and discoloration is seen for accumulated doses of approximately 106 Grays [223]. Interfacial stresses may arise, causing delamination and failure.

The radiation resistance of other materials has been discussed in [218,220,221,224]. The delamination at the interfaces such as piezo element–backing, piezo element–matching layer, or piezo element–wear plate can occur. Several works suggest a significant degradation of the materials used in the matching layer and wear plate such as blistering, bubbling, discoloration, and deformation seen during ultrasonic inspections of CANDU reactor components [69]. Therefore, some transducers incorporate design changes to avoid materials susceptible to radiation. Endurance test results with respect to organic materials used in ultrasonic piezoelectric transducers [225] suggest radiation-resistant adhesives to maintain the bond line integrity of the matching layer. Alternative methods such as dry bonding and pressurized dry coupling, pressurized liquid coupling serve as an alternative option, but they can be expensive and unreliable [217,226,227].

Debonding of the backing layer from the piezoelectric element causes a distortion of the signal by excessive ringing. A reduction of bandwidth and poor time resolution due to ultrasonic echoes can appear. Such failures such as blistering of the matching layer, delamination and detachment of backing layer discoloration, water leakage, loss of bandwidth, efficiency drop, and loss of sensitivity of 3–13 dB without failures were seen for a gamma dose of 1–2 MGy [228,229,230]. Periodic recalibration and recovery by post-irradiation biased annealing [223,231,232,233,234] can be carried out for mitigating minor damage to the piezoelectric element. However, the damage in other components of the transducer may render the transducer inefficient. Lead metaniobate is employed as the piezoelectric element for its superior temperature stability [231]. The PCB Piezotronics [235] provides piezoelectric devices for accumulated gamma doses of approximately 1 MGy. Researchers have proposed the use of faceplates and matching layers after 0.46–0.65 MGy of absorbed dose for stability after fabrication [236]. Plastics used as backing, a 1/4-wave acoustic impedance matching layer, and protective face need to have strong radiation resistance for which several epoxy systems are suitable [220]. The thermal mismatch between transducer layers can be avoided during the design stage by selecting materials with closely matching thermal expansion coefficients.

## 3. Special Acoustic Matching Layer Materials

The unsuitability of high impedance PZT materials for hydrophone and biomedical applications require matching layers. High to very high-frequency applications require material properties that are commercially unavailable and have called for the development for alternate materials. In this section, we discuss several novel materials other than the one reported in Section 1.3.

### 3.1. Composites and Nanocomposites as a Passive Matching Layer

The 0–3 nanocomposites based on Al_2_O_3_ [237], CeO_2_ [238], SiO_2_ [239], TiO_2_ [240], and Ag [212] at high frequencies 10–100 MHz have nonuniformity and high attenuation (>20 dB/mm at center frequency) caused by particle scattering. A large volume fraction (>40%) of particles is difficult to be embedded in the matrix due to the wetting problem. Changing the type of connectivity such as silicon–epoxy 1-3 and 2-2 composite matching layers fabricated by deep reactive ion etching serve as alternatives [187,241,242]. Table 5 lists various materials used as passive acoustic matching layers in piezoelectric transducers and compares their performance.

**Figure 9:**
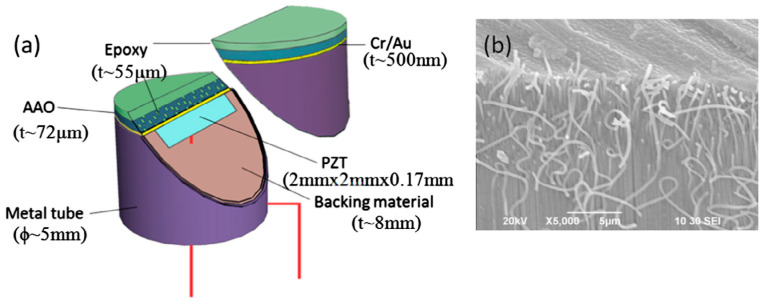
(**a**) Schematic diagram of the designed PZT-5A transducer and (**b**) cross-sectional SEM of AAO–epoxy 1-3 composite. Reprinted from [157], Copyright (2016), with permission from Elsevier.

Fang et al. [157] used anodic aluminum oxide–epoxy 1-3 composite matching layers and designed two-layer matching as per transmission line method using the KLM model (see Section 2.2.1 for the method). The acoustic impedance of the PZT-5A ceramic and load medium 1.5 MRayls for the human body was matched. The calculated acoustic impedance of the first and second layers was 9.1 MRayls and 2.4 MRayls, respectively. An AAO–epoxy composite as the first matching layer and pure epoxy (Epotek 301) as the second matching layer with properties listed in Table 4 were chosen. The backing material with high attenuation (−50 dB in the backing thickness at center frequency) widens the bandwidth by absorbing the radiated ultrasonic energy and reducing the ringing. It reduced the sensitivity [77,243] for which the bandwidth and signal amplitude was enhanced by a backing layer made of a mixture of Epotek 301 epoxy, tungsten powder, and microbubbles with properties [244] listed in Table 4. The KLM model-based simulation software Piezo CAD was used to predict the performance, which revealed a center frequency of 11.2 MHz with a bandwidth (−6 dB) of 70%. The PZT-5A ceramic with an active area of 2.0 × 2.0 mm^2^ and a thickness of 170 μm was used as the active element of the transducer. The bottom electrode of this active element was bonded to a copper wire terminated with a BNC connector. Then, the mixture of tungsten powder/micro bubbles/Epotek 301 was cast on the bottom electrode and filled the metal housing as the backing layer. Finally, the double matching layers were bonded to this top electrode under an external pressure with about 20,000 Pa by pressing a piece of metal as shown in Figure 9. Experimentally obtained voltage response and spectrum measurements showed a broad bandwidth of 68% (−6 dB) and a two-way insertion loss of −22.7 dB estimated from Equation (20) and Equation (26), respectively.

**Figure 10:**
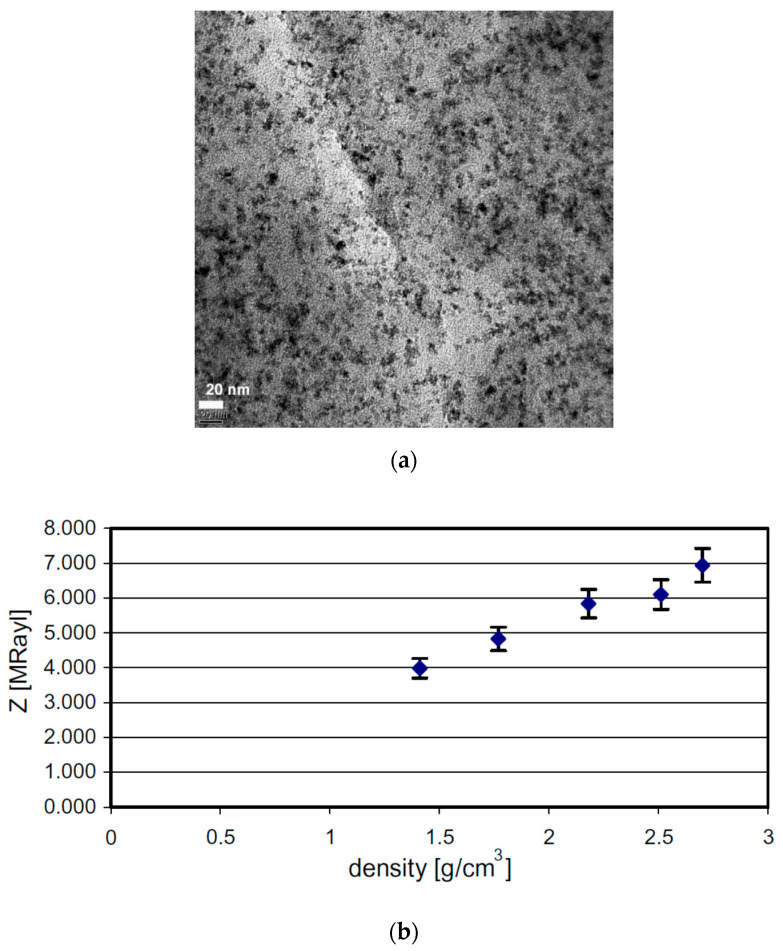
(**a**) A TEM picture of the epoxy matrix embedded with cerium oxide particles (10 wt % CeO_2_). (**b**) Variation of acoustic impedance of cerium oxide/epoxy functionalized organic–inorganic hybrid polymer nanocomposite. Reprinted from [238], Copyright (2010), with permission from Elsevier.

Novel thermoplastic nanocomposite foams are being considered for air-coupled ultrasonic applications for their superior performance as well as cost-effective manufacturing. A chemical blowing agent (CBA) liberates a blowing gas (CO_2_, H_2_O, NH_3_) in the foaming process under thermal decomposition [245,246]. Instead of relatively light Al_2_O_3_ (ρ = 3.94 g/cm^3^) particles, Tiefensee et al. [238] used CeO_2_ particles (ρ = 7.13 g/cm^3^) to reach an acoustic impedance of 6.8 MRayl for coupling from most piezoelectric ceramics into water. The density of the matching layer between 2.0 and 3.0 g/cm^3^ was needed, for which 10 nm and 15 nm CeO_2_ nanoparticles served the purpose. Upon curing with an epoxy, a silane nanocomposite was formed with an inorganic particle network. A TEM picture of a nanocomposite with 10 wt % cerium oxide particles prepared with microtome cutting is shown in Figure 10a. The particles were homogeneously distributed in the matrix that had a sound velocity of 2100 m/s. Materials with 34 wt % and 75 wt % nanoparticle content (9–37 vol.% respectively) were prepared to vary the acoustic impedance as it varied the density, as shown in Figure 10b. The acoustic impedance varied between 4 and 7 MRayl linearly with the density and with an attenuation of around 0.5 dB/µm.

Backing materials damp out transducer ringing by absorbing the acoustic energy from the backward propagating energy and from unwanted shear waves. This also shortens the pulse duration and broadens the bandwidth, reducing the transducer sensitivity. Backing materials should have good acoustic coupling to the piezoelectric element. Similar acoustic impedances will transmit acoustic waves into the backing materials without reflection from the interface. The backing material with high attenuation property also eliminates any energy from reflecting to the piezoelectric element. Usually, a single layer of backing serves these purposes. Epoxy resin loaded with tungsten powder is the frequently used backing material. A wide range of acoustic impedance values results by changing the content of tungsten powder (101 MRayl) in epoxy resin (3–4 MRayl) [101].

### 3.2. Acoustic Metamaterials and Metasurfaces

Traditional quarter-wavelength matching improves the energy transmission at the operating frequency with the proper matching material. These techniques lead to a narrow pass-band window of operation for which they are unsuitable for short pulse operation (broadband) to obtain good resolution for applications such as medical diagnostics, nondestructive evaluation, and underwater acoustics. New emerging ferroelectric single-crystal materials such as PMN–PT [78,247,248,249,250,251,252,253,254,255] have an exceptional piezoelectric performance with 5 times higher strain energy densities and significantly higher electromechanical couplings than other piezoelectric ceramics [249,250]. Recently, materials such as phononic crystals and acoustic metamaterials have resulted in many novel applications such as sound blocking [256,257], imaging [258,259], acoustic cloaking [260,261,262,263,264], absorption [265,266,267,268,269,270,271], multiple exceptional point [272,273], and topological acoustics [274,275,276,277,278,279,280]. Phononic crystals block the wave propagation by acoustic band gaps. Acoustic metamaterials are made of periodic artificial structures referred as “meta-atoms”. Each meta-atom has a size larger than the conventional atom and much smaller than the radiated wavelength and these are thereby used for the deep control and manipulation of acoustic waves [281]. Acoustic metamaterials have zero to negative refractive index offering new possibilities for the control of sound at the subwavelength scale [282]. Acoustic metamaterials exhibit negative mass density [253,283,284,285], negative bulk modulus [252,286,287,288], negative shear modulus [289], extreme anisotropy [290,291,292], and coiling behavior [293,294]. Acoustic metamaterials involve the collection of subwavelengths called unit cells or metamaterial bricks. These are characterized by effective mass density and bulk modulus.

Traditional approaches for acoustic impedance matching using metamaterials include the use of Fabry–Pérot resonances [295,296,297] and gradient index structures. The Fabry–Pérot resonance approach is sensitive to geometric size and frequency, whereas the gradient index approach requires complex geometries. Some of these shortcomings have been overcome by using artificial acoustic materials [298,299,300,301,302,303,304]. Matching layers with continuously changing acoustic impedance, especially with exponential variation, provide satisfactory transmission and reflection properties [305]. Impedance matching with phononic crystals and acoustic metamaterials holds the possibility of near-unity transmittance [255,299,301,305,306].

**Figure 11:**
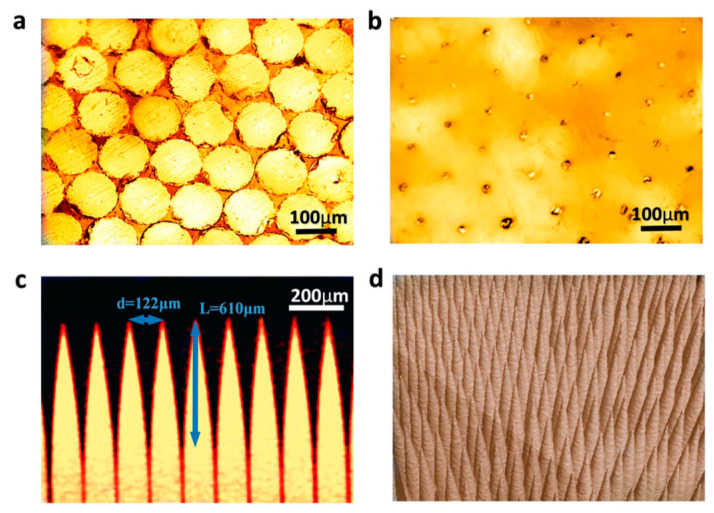
Pyramidal cone metamaterial matching layer showing (**a**) hexagonal close-packed silica columns with maximum acoustic impedance nearing silica, (**b**) top side of the matching layers showing the tips of the silica fibers with the lowest acoustic impedance, (**c**) the cross-section of the metamaterial matching layer, and (**d**) the 3D topography showing the pyramidal cone arrays. Reproduced from [307], an open-source article licensed under a Creative Commons Attribution 4.0 International License.

**Figure 12:**
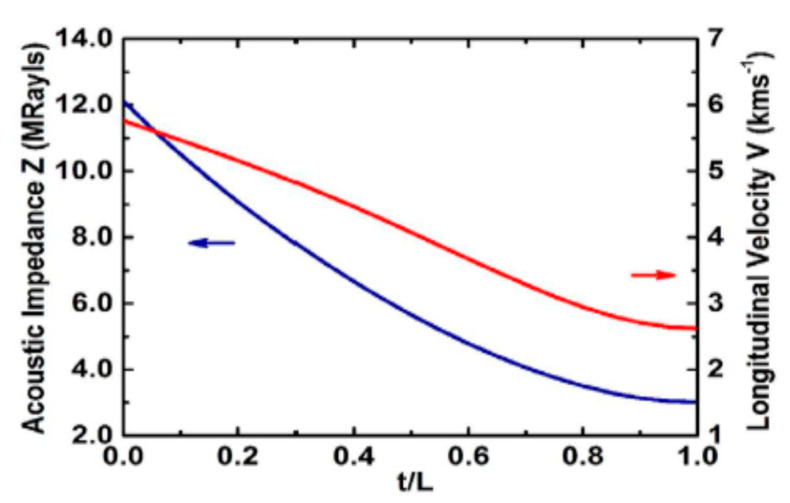
A continuous and monotonic distribution of the equivalent acoustic impedance along the thickness direction of a metamaterial matching layer. Reproduced from [307], an open-source article licensed under a Creative Commons Attribution 4.0 International License.

Li et al. [307] proposed a 1 mm thick gradient layer matching scheme to provide a solution for the unsolved problem of an efficient broadband acoustic impedance matching scheme. The scheme consisted of an anisotropic cone-structured acoustic metamaterial matching layer with periodically arranged subwavelength silica–epoxy composite unit cells. The volume fraction of the silica cone was designed to decrease away from the piezoelectric material, as shown in Figure 11. It provided gradually changing acoustic impedance 11.4–3.0 MRayls along the direction of wave propagation. Fabrication was carried out by etching the peeled silica optical fiber bundles with hydrofluoric acid solution providing a −6 dB percentage bandwidth of approximately 100% around the resonant frequency. The monotonic and continuous acoustic impedance variation along the thickness is shown in Figure 12, and it varies as per the equation for 1–3 composites given by

(44) $$Z=\sqrt{\left\{n\left[C_{11}^{\prime }-\frac{2\left(1-n\right){\left(C_{12}^{\prime }-C_{12}\right)}^{2}}{n\left(C_{11}+C_{12}\right)+\left(1-n\right){\left(C_{11}^{\prime }+C_{12}^{\prime }\right)}^{2}}\right]+\left(1-n\right)C_{11}\right\}/\left[\rho n+\rho^{\prime }\left(1-n\right)\right]}\;,$$

where $C_{ij}$ and $C_{ij}^{\prime }$ (i,j = 1,2) are the elasticity coefficients of the metamaterial, ρ and $\rho^{\prime }$ are the densities, n is the volume fraction of silica given by

(45) $$n=\frac{\pi }{2\sqrt{3}}{\left(1-\frac{t}{L}\right)}^{2}.$$

Equation (45) represents the variation of n with the distance t along the cone with the length L. With such a broadband window, ultrasonography systems can utilize the full potential of single-crystal piezoelectric materials.

Most of the matching techniques are suitable for a normal incidence of waves. Perfect acoustic absorbers require wide-angle impedance matching for applications in noise control and stealth technology. Imaging enhances with wide-angle matching. Such abilities are demonstrated using ultra-transparent media for use in photonics [308,309]. Liu et al. [309] proposed a wide-angle matched acoustic metamaterials using a spatially dispersive effective medium theory by matching silicone rubber of huge impedance mismatch with water. One and two-dimensional acoustic structures were matched to achieve near 100% transmission. To match the impedance of the effective medium with water, $Z_{eff}$ = $Z_{w}$ the conservation of the tangential component of wave vector at the interface $k_{w,y}=k_{y}$ gives the condition of impedance matching as

(46) $$\frac{\rho_{eff,x}\rho_{eff,y}}{\frac{\omega^{2}\rho_{eff,y}}{K_{eff}}-k_{y}^{2}}=\frac{\rho_{\omega }^{2}}{k_{\omega }^{2}-k_{y}^{2}}\;,$$

where $k_{w}=\omega \sqrt{\rho_{w}/K_{w}}$, $Z_{w}=\frac{\rho_{w}}{k_{w,x}}\omega$ is the wave impedance of media (water), $Z_{eff}=\frac{\rho_{eff,x}}{k_{x}}\omega$ is the wave impedance of effective medium, and $k_{w,x}$ and $k_{w,y}$ are the wave vectors along the x and y directions respectively related to $k_{w}$ by dispersion $k_{w,x}^{2}+k_{w,y}^{2}=k_{\omega }^{2}$. The dispersion of effective medium is given by $\frac{k_{x}^{2}}{\rho_{eff,x}}+\frac{k_{y}^{2}}{\rho_{eff,y}}=\frac{\omega^{2}}{K_{eff}}$, where $\rho_{eff,x}$ and $\rho_{eff,y}$ are the effective mass density along the x and y directions. The wavenumber of the acoustic matching layer is given by $k=k_{x}\hat{x}+k_{y}\hat{y}$. For a wide range of incident angles, the shifted spatial dispersion is given by $\frac{{\left(k_{x}-p\right)}^{2}}{q}+k_{y}^{2}=k_{\omega }^{2}$ [149,308,310], where p denotes the displacement from the Brillouin-zone center, and q determines the ratio of the $k_{y}$ and $k_{x}$ axes of the ellipse. Further, from above Equation (46), the spatially dispersive parameters are obtained as

(47) $$\rho_{eff,x}\left(k_{y}\right)=\pm \frac{\left[p\pm \sqrt{q\left(k_{w}^{2}-k_{y}^{2}\right)}\right]\rho_{w}}{\sqrt{k_{w}^{2}-k_{y}^{2}}}$$

(48) $$\frac{\omega^{2}}{K_{eff}\left(k_{y}\right)}-\frac{k_{y}^{2}}{\rho_{eff,y}\left(k_{y}\right)}=\pm \left[p\pm \sqrt{q\left(k_{w}^{2}-k_{y}^{2}\right)}\right]\frac{\sqrt{k_{w}^{2}-k_{y}^{2}}}{\rho_{w}}\;.$$

The above Equations (47) and (48) presents a solution for $\rho_{eff,x}$ and $\rho_{eff,y}$ that satisfies impedance matching.

**Figure 13:**
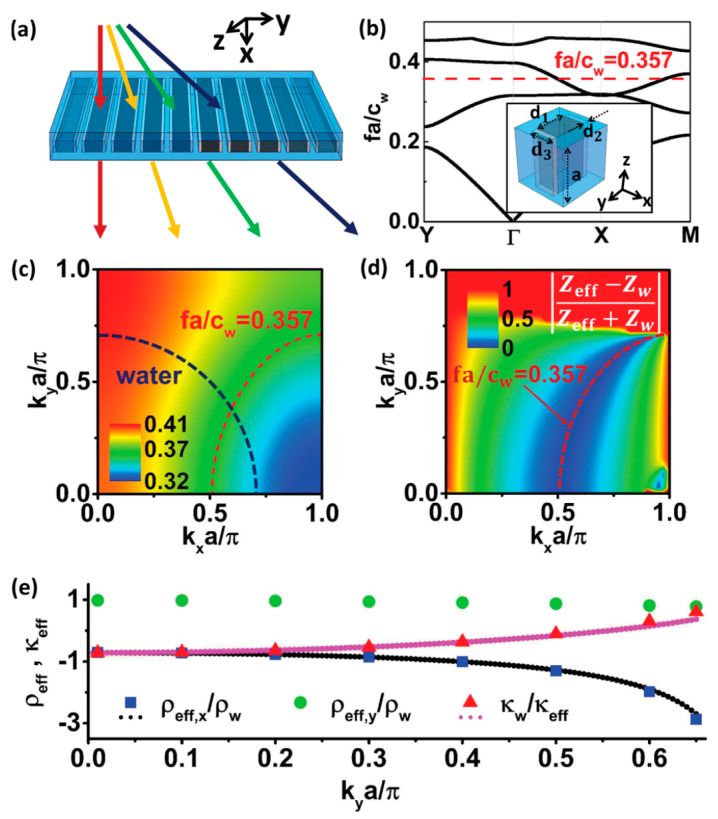
(**a**) Schematic of a 2D impedance-matched acoustic matching layer. (**b**) Band structure where the red dashed line denotes the central frequency $f_{a}/c_{w}$ = 0.357 and inset showing the unit cell. (**c**) The equal-frequency contour of the third band. The red and blue dashed lines denote the equal frequency contours of the acoustic matching layer and water at $f_{a}/c_{w}$ = 0.357, respectively. (**d**) Impedance difference between the acoustic matching layer and water. (**e**) Effective parameters $\rho_{eff}$ and $K_{eff}$ of the 2D acoustic matching layer. Reprinted with permission from [309], DOI: https://doi.org/10.1103/PhysRevMaterials.2.045201, Copyright (2018) by the American Physical Society.

**Figure 14:**
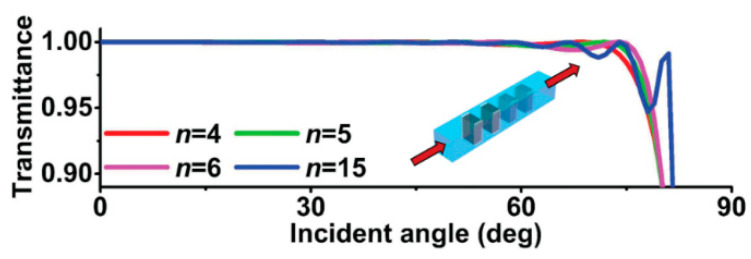
Incident-angle-dependent transmittance of acoustic waves propagating through an acoustic metamaterial matching layer with *n* = 4, 5, 6, 15 unit cells. Reprinted with permission from [309], DOI: https://doi.org/10.1103/PhysRevMaterials.2.045201, Copyright (2018) by the American Physical Society.

Unlike metamaterials that are generally referred to 3D structures, acoustic metasurfaces are 2D metamaterials designed from thin material layers of subwavelength thickness to manipulate sound [311]. The unit cell of the 2D acoustic matching layer was constructed by two kinds of porous silicone rubber rods periodically aligned as shown in Figure 13a. The periodic structure formed a square lattice in the *y–z* plane. The silicone rubber rod having $\rho_{S1}$ = 1039.5 kg/m^3^ and $c_{S1}$ = 679.4 m/s is sandwiched by the silicone rubber rod $\rho_{S2}$ = 1033.8 kg/m^3^ and $c_{S1}$ = 246.4 m/s, as shown in Figure 13b. The filling ratio of the air-filled porous silicone rubber is altered for property tuning [312]. The parameters shown are $d_{1}$ = 0.6*a*, $d_{2}$ = 0.1*a*, and $d_{3}$ = 0.4*a*. Here, *a* is the lattice constant. The band structure is presented in Figure 13b. The equal-frequency contour of the third is shown in Figure 13c. The shear modulus of the silicone rubber is smaller than the bulk modulus showing negligible transverse modes [313,314]. The equal-frequency contour is an ellipse centered at the X point with a shift in the $k_{x}$ direction implying spatial dispersion within the matching layer to obtain a wide-angle impedance-matching effect. The impedance difference between the matching layer and water showed a very small impedance difference around the central frequency for a large range of $k_{y}$ (See Figure 13d), allowing broadband and wide-angle impedance matching between the matching layer and water. The effective parameters further coincided very well (see Figure 13e) over a large range of $k_{y}$, indicating a wide-angle impedance matching within the matching layer with spatial dispersion. The broadband and wide-angle impedance matching were further studied by transmittance for acoustic waves passing through the matching slab consisting of *n*(= 4, 5, 6, 15) unit cells in the *x*-direction. Figure 14 shows almost 100% transmission for all θ < 75°. 

Acoustic impedance matching was done by Memoli et al. [315] using 3D metamaterial bricks to obtain a focused beam by fabricating quantal metasurface. Jahdali and Wu [302] reported the design of acoustic lenses tailored by acoustic metasurfaces comprising rigid thin plates with periodically distributed subwavelength slits. The impedance of the lens was matched with background media, and the focusing capability was demonstrated.

## 4. Acoustic Matching for Specific Transducer or Sensor Type

### 4.1. Very High-Frequency Ultrasonic Transducers

High-frequency (>20 MHz) transducers utilizing piezocomposites have been extensively studied for ultrasonic imaging [128]. Piezoelectric composites utilizing piezo rods embedded in the passive polymer matrix have received great attention. Shen et al. [316] developed 1–3 type (Na,K)NbO_3_ based Pb-free piezocomposites for high-frequency transducer applications. Spark plasma sintering was used to prepare Li/Ta-modified KNN ceramics with an optimized nominal composition of (Na_0.535_K_0.485_)_0.95_Li_0.05_(Nb_0.8_Ta_0.2_)O_3_ (NKLNT). A 1–3 composite based on NKLNT was fabricated using the dice-fill method [317]. Good electrical properties were achieved with a high piezoelectric constant ($d_{33}$ = 5140 pC/N), low acoustic impedance (Z = 56.6 MRayls), high electromechanical coupling coefficient ($k_{t}$ = 0.655), reduced dielectric constant ($\epsilon_{r}$ = 302), piezoelectric voltage coefficient ($g_{33}$ = 52.4.4 × 10^−3^ m^2^/C), and mechanical quality factor (Q = 18). A very broad bandwidth of 89.7% at −6 dB and at 29 MHz was developed.

**Figure 15:**
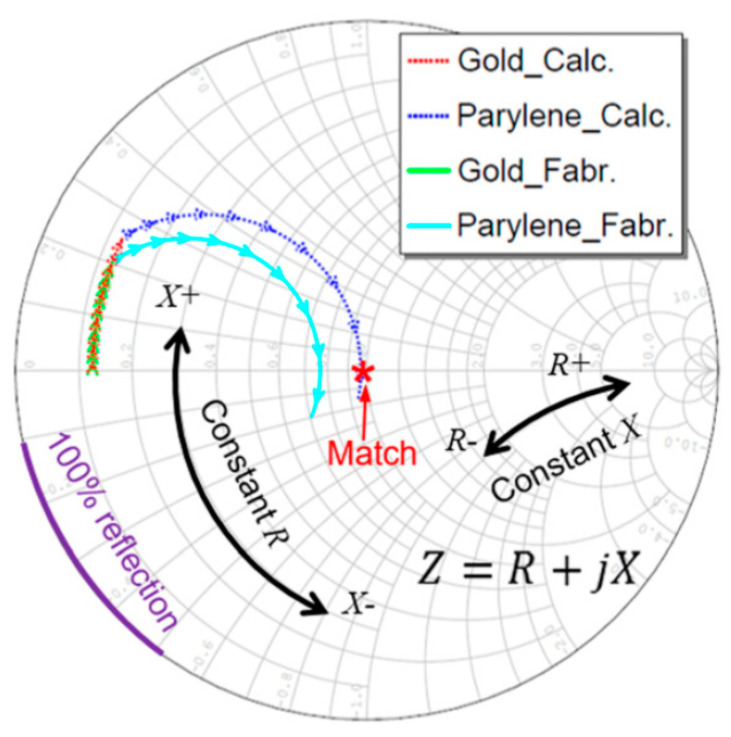
Smith chart showing the acoustic impedance loci of transducer normalized to 12.13 MRayl and obtained using the transmission line model (Calc.) and KLM-based spring-mass model (Fabr.). Reprinted from [154], with the permission of AIP Publishing.

**Figure 16:**
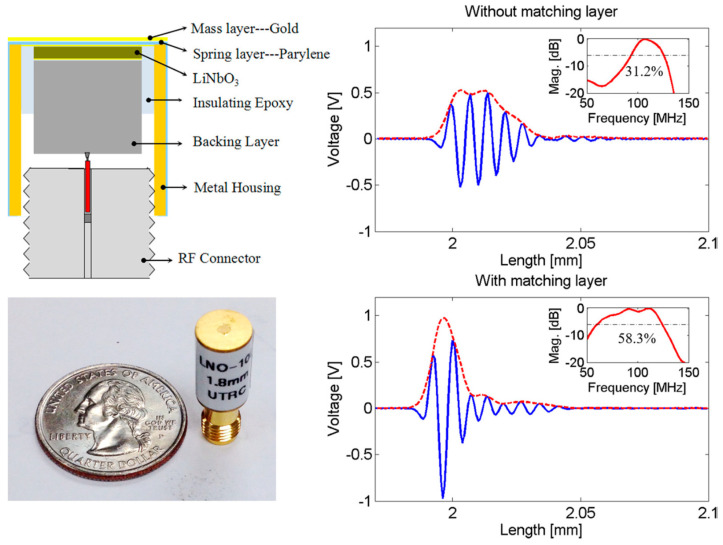
Schematic and photo of the 100 MHz LiNbO_3_ transducer with matching layers shown on the left. A comparison is made on the right showing the pulse–echo experimental response of the transducer before and after the matching layers deposition on the top surface. Reprinted from [154], with the permission of AIP Publishing.

Requirements of satisfying specific acoustic impedance and precise thickness have been problematic, especially for broadband very high-frequency ultrasonic transducers (>100 MHz). Difficulty in achieving ultra-thin matching layers for a high-frequency range for the quarter-wavelength matching method has led to the use of the mass-spring approach and transmission line approach. These approaches have been discussed in Section 2.2 and Section 2.3, respectively. Fei et al. [154] used these approaches to match the acoustic impedance of a 100 MHz transducer with water. The loci of the transducer impedance shown in the Smith chart of Figure 15 is normalized to 12.13 MRayl, indicating optimization lines for the transmission line as ‘Calc’ and the KLM spring-mass model as ‘Fabr’. Both methods estimated an impedance of 12.13 MRayl for 250 nm gold thickness and 2.5 µm parylene thickness. The shifting of impedance from edge to center indicates the matching of the complex value of acoustic impedance and reflection coefficient using the two layers. The continuous variation of the impedance during optimization using the Smith chart leads to the capability of impedance matching without strict dependence on the specific impedances of the materials. Figure 16 shows the fabricated transducer and its pulse–echo response obtained with and without matching layers. Parylene layer was vapor-deposited, and the gold layer was sputtered. The amplitude was enhanced by 68.6%, whereas −6 dB bandwidth was enhanced from 30.2% to 58.3%. Such acoustic impedance-matching techniques can be further developed to suit modern composite acoustic devices such as High-tone Bulk Acoustic Resonators (HBAR) and Solid Mounted Resonator (SMR) operating at microwave operational frequencies (20 GHz) [318,319,320,321]. These devices of thin film piezoelectric materials have interesting frequency response characteristics concerned with the acoustical performance and are of great importance as sensors.

### 4.2. Piezoelectric Micromachined Ultrasonic Transducers

Microelectromechanical systems (MEMS), fabricated by means of micromachining technology, have advantages including small size and batch fabrication with low manufacturing cost, flexibility in frequency ranges, and high resolution. MEMS ultrasound devices are commonly known as micromachined ultrasonic transducers (MUTs) with piezoelectric form (pMUT). The pMUTs have linear drive responses, high vertical deformation, and require lower driving voltage, but they suffer from lower electromechanical coupling factors. Muralt and Baborowski [322] discussed the best possible coupling coefficient of a piezoelectric heteromorphous membrane amounting to typically 60% in bulk PZT, since half of the vibrating structure is passive. Thus, the coupling coefficient is 10% to 20% less than $k_{33}$ used in bulk transducers. They further discussed the applications that need high coupling. Akhbari et al. [323,324] discussed the electric equivalent model of pMUTs, which can be readily used for acoustic impedance layer-matching designs. Lee et al. [325] used collagen-filled deep reactive ion etched back-side hole for acoustic impedance matching for 2D pMUT arrays and a Petri dish on a fluorescence microscope. The targeted application was cell stimulation. Akasheh et al. [326] altered the impedance of micromachined piezoelectric films to match with the acoustic impedance of water for use in AC acoustic transducers.

### 4.3. Air and Liquid Coupled Transducers

Airborne transducers have become valuable in developing systems related to robotics [327], material characterization, in situ monitoring, acoustic microscopy, nondestructive evaluation [328,329,330], human–computer interaction [331,332,333,334,335,336], ultrasonic vortex generation [337], tactile [338], and metrology [339]. Unlike liquid-coupled ultrasonic transducers, air-coupled ultrasonic transducers are clean and easy to use [335,340,341,342,343]. It also avoids cleaning or drying and possible moisture damage to the parts being inspected [344]. Therefore, the noncontact NDE is fast and saves inspection time and cost in an industrial setting [345]. The noncontact transducers are often bulky when compared to contact transducers and are very carefully designed to achieve high power efficiency and signal quality for industrial use. They suffer either from the limitation in the availability of materials for effective impedance matching and their associated attenuation losses. The high difference of acoustic impedance of a piezoelectric ceramic element $Z_{A}$ = 30 MRayl and air $Z_{B}$ = 0.000425 MRayl requires multiple matching layers. Even transducers coupled with water that have $Z_{A}$ = 1.5 MRayl need multi-layer matching. Multi-layer matching causes internal reverberations of the transmitted wave. The ratio of transmitted to incident acoustic energy from the matching layer to the air is given by

(49) $$\Upsilon_{t}={\left(\frac{T_{1}T_{2}e^{-\alpha l}}{1-R_{1}R_{2}e^{-2\alpha l}}\right)}^{2}\frac{Z_{B}}{Z_{A}}\;,$$

where $T_{1,2}=2Z_{A,M}/\left(Z_{A,M}+Z_{M,B}\right)$ represents the ratio of transmitted to incident wave amplitude and $R_{1,2}=\left(2Z_{M,B}-Z_{A,M}\right)/\left(Z_{M,B}+Z_{A,M}\right)$ represents the ratio of the reflected wave amplitude to the incident wave amplitude. The loss in dB is given by

(50) $$\Upsilon_{t}\left(\mathrm{dB}\right)=10\log(\Upsilon_{t}).$$

Thus, the attenuation in the matching layer is regarded as a one-way insertion loss, regardless of the other components of the transducers and excitation. Therefore, the loss that is dependent purely on the material is of great importance in the development and selection of special materials for matching layers. Gomez Alvarez-Arenas [176] studied the optimum impedance of the matching layer required for materials having different attenuation loss coefficients. The performance had a little dependency on attenuation loss for water-coupled transducers, as most of the energy was transmitted within the first reflection. The estimated optimum value for an air-coupled transducer was 0.12 MRayl, and that for a water-coupled transducer was 6.4 MRayl. An important aspect of the attenuation coefficient is the variation with frequency. If the variation is linear with frequency, such as that seen in constant-Q materials, the insertion loss is independent. However, certain materials such as porous solids especially used as the matching layers in air-coupled ultrasound have variation described by a power law with 0.5–4 as a value for the exponent. The power law is given by

(51) $$\Upsilon_{t}=\Upsilon_{0}f^{\beta -1}\;,$$

where $\Upsilon_{0}=\alpha_{0}v$. Complex mechanisms such as viscosity, thermal dissipation, friction, viscoelasticity, and multi-phase scattering give rise to such a power law, which is difficult to theoretically establish [346,347]. Air-coupled transducers require an outer matching layer to possess 0.04–0.3 MRayl with $\Upsilon_{t}$ = 0.14 Np. In addition, broadband transducers require less or a linear dependency of attenuation coefficient with frequency. Such a requirement is difficult to achieve. Silica aerogel has an acoustic impedance of 0.1 MRayl and $\Upsilon_{t}$ = 0.06 Np, but it is difficult to machine [348,349,350,351]. The silicon-loaded microspheres developed have a tradeoff with low impedance 0.3 MRayl and low attenuation $\Upsilon_{t}$ = 0.6 Np.

An interesting approach is the use of a porous membrane, which was reported for the first time using polyolefin material [352] with $\Upsilon_{t}$ = 0.276 Np. Cellulose nitrate membrane showed better properties with $Z_{M}$ = 0.12 MRayl and $\Upsilon_{t}$ = 0.25 Np [353,354]. Polyethersulphone and nylon membranes with a pore size of 0.1 µm exhibit even lower attenuation of 0.044 Np and 0.14 Np respectively, with a proper acoustic impedance of 0.1–0.313 MRayl. Various other membrane filters have been reported to have $Z_{M}$ = 0.08–0.63 MRayl and $\Upsilon_{t}$ = 0.1–0.6 Np with λ/4 and a resonant frequency of 0.3–2 MHz.

**Figure 17:**
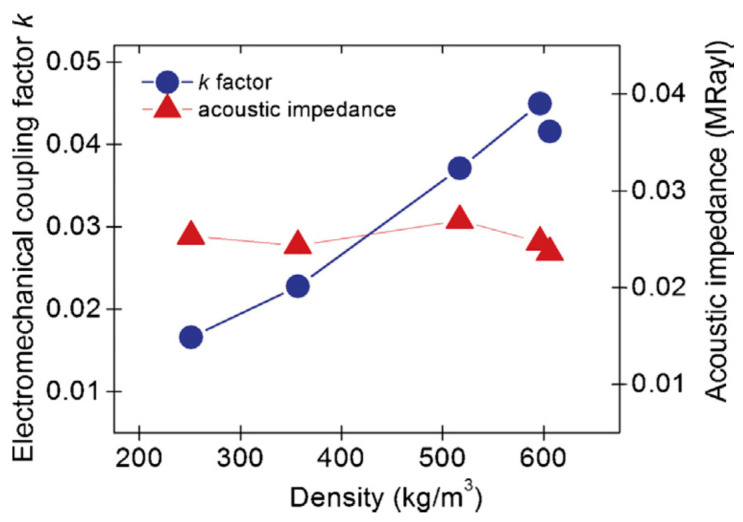
Acoustic impedance and electromechanical coupling factor *k* (obtained from the fit of dielectric spectra) for the given sample density range. Reprinted from [359], Copyright (2015), with permission from Elsevier.

Instead of passive matching layers, active matching layers have been used widely in air-coupled ultrasound. Galbraith and Hayward [355] proposed piezoelectric membranes made of poled PVDF as air-coupled ‘‘hydrophones’’ or ‘‘aerophones,’’. Heterogeneous cellular polymer ferroelectrets have shown strong piezoelectricity [356,357,358]. Sborikas et al. [359] used cellular polypropylene (PP) films as a piezoelectric element, which has an acoustic impedance of 0.024–0.027 MRayl closely matching with an acoustic impedance of air (400 Rayl) to develop an air-borne transducer for the inspection of fabrics. Acoustic impedance was changed by obtaining films of different density 251–606 kg/m^3^ using expansion and thermal treatment-based fabrication, as shown in Figure 17. The matching of acoustic impedance eliminated the requirement of matching layers. They obtained a bandwidth of 35% at −6 dB with a resonance frequency around 150 kHz. The acoustic transmittivity was found to be 6 mPa/V. The highest k = 0.045 was seen at a density of 596 kg/m^3^, which is lower compared to piezoceramics. However, the figure of merit (FOM) [128,140] was found to be higher for PP films, which considers both k and Z. Polymer ferroelectrets have also been considered to develop a water-coupled transducer [360], as the acoustic impedance of the ferroelectret film is closer to the acoustic impedance of water (Z = 1.48 MRayls). de Medeiros et al. [361] proposed an ultrasonic hydrophone based on a piezoelectret made of fluoroethylene–propylene films Z = 0.03 MRayl [362,363,364,365,366,367] to be used in ultrasonic imaging and vibroacoustography [368,369]. Nylon Z = 2.0–2.3 MRayl was employed as a matching layer to match the acoustical impedance with water.

To acoustically match piezoelectric crystal with air, the acoustic impedance of piezoelectric crystal needs to be around 80 kRayl. Such a solid material is difficult to find among natural solid materials. Highly porous solid foam with a compliant web can meet the density and stiffness requirement but gives rise to a narrow frequency range of operation. Scattering of the wave around cell boundaries needs to be avoided to prevent de-phasing. Such a material is silica aerogel, which can be used between piezoceramics and air as a matching layer [349]. Toda [370] proposed a different type of layer consisting of a thin membrane placed at a distance of a piezoelectric transducer via a perforated film showing a significant improvement (up to 10 dB) in a narrow frequency band of approximately 40 kHz.

Piezocomposites improve acoustic impedance matching when compared to bulk piezoceramics but in a rather narrow frequency band [353,371,372,373]. Even some sophisticated matching layer(s)-based transducers [176,346,374] using silica aerogels (0.015 MRayl) and thin porous filtration membranes work in a relatively narrow bandwidth. A porous layer was combined with a low-density rubber as layers, resulting in a better amplitude enhancement falling only 20 dB from a maximum at 0.50–2.0 MHz [354]. Bovtun et al. [375] used ferroelectric films by integrating them with high impedance amplifiers for acoustic wave transmission in air.

Castaings et al. [376] used 1–3 composite active elements to develop matched air-coupled transducers for the nondestructive evaluation of composite laminates. Piezo-ceramic rods embedded in a polymer provided matching to minimize the enormous acoustic impedance mismatch between air and the transducer. Mechanical impedance of the transducers was controlled by adjusting the ceramic/polymer volume fraction. This was extremely important, as the response is nearly zero with the unavoidable loss due to the reflection between air and composite laminate [377]. The reflection coefficient at the top surface of the component approaches unity, and transmission of the reflected wave from the defect has the transmission coefficient nearly equal to zero, and thus very little energy is transmitted back to the receiver. The signature from defect being much smaller than the initial specular reflection from the surface makes it impossible to use normal incidence single-sided inspection, as the signals of interest will be overloaded by the specular reflection. Therefore, oblique incidence testing was considered.

**Figure 18:**
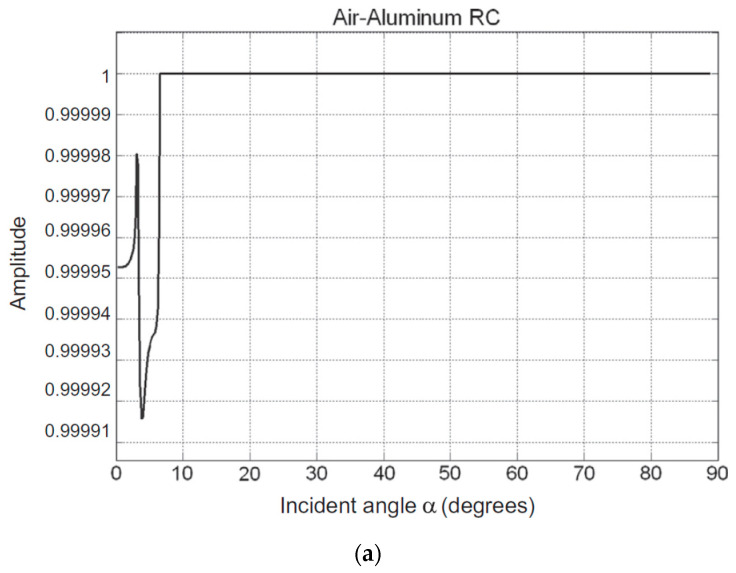

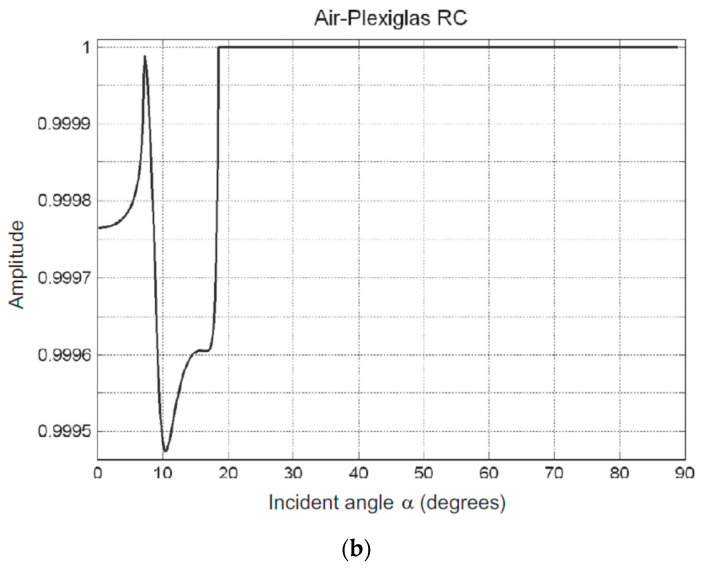
Reflection coefficient of an (**a**) air–aluminum interface and (**b**) air–plexiglass interface. Reprinted from [335], Copyright (2014), with permission from Elsevier.

Chimenti [335] considered the study of transduction performance with incidence angle for an application involving the testing of concrete. Aging concrete infrastructure was estimated to cost almost 3.6 trillion dollars for quality reinstatement in 2017 [378]. Durability is mostly affected by microcracking, which increase with time [379]. Nondestructive assessment of these structures is limited to small structures using noncontact ultrasound [380,381]. Contact-based ultrasound offers the capability to assess large structures but is impractical to evaluate large infrastructure. Inspection engineers are left with the choice to improve noncontact ultrasound techniques. Noncontact air-coupled ultrasound offers through-thickness assessment by enabling rapid data collection and a real-time display of intuitive scan results. The major hurdle in the development was imposed by large acoustic impedance mismatch from the transducer (Z = 36.15 MRayl) to air (Z = 0.00042 MRayl) and then from air to concrete interfaces (Z = 8.36–11.13 MRayl). Multiple reflections reduce the received energy drastically. The resulting reflection coefficient from PZT to air is nearly –1 (−0.999, see Equation (17) in Section 1.4) calling for the utilization of acoustic impedance matching layers. Chimenti [335] operated the transducer at its electrical resonance and receiver at its mechanical resonance to maximize their efficiency. The variation of reflection coefficient with the angle of incidence of the transmitted beam is shown in Figure 18 for the air–aluminum interface. It shows a very small range of critical angles and a minimum value of reflection coefficient at the first critical angle, which is still near unity. The onset of total reflection is within 8°. Even for an air–plexiglass interface, the onset of total reflection occurs before 20°. Acoustic matching layers for air-coupled and liquid-coupled ultrasonic transducers have been also proposed for applications in the food industry such as drying, apple firmness, defoaming, and decontamination [382,383,384,385].

### 4.4. Phased Array Transducers

Ultrasonic arrays are used in sonar and medical imaging due to their acoustic beam focusing and steering capabilities [386,387]. Properties such as non-diffraction, self-bending, twisting, and focusing of the wave front are of main interest [388,389,390]. Lau et al. [159] proposed a double λ/8 matching layer scheme for acoustic impedance matching for a 16-element phased array transducer with PMN–PT single crystal (72 mol% of PMN) active elements. The double λ/8 layers resulted in a quarter λ/4 matching layer design. An element center-to-center spacing was 0.28 mm. A highly attenuating backing with matched acoustic impedance comprising two types of backing listed in Table 4 as tungsten powder, microbubbles, and epoxy composite was considered. The acoustic impedances of two matching layers were estimated as per the two-layer quarter wavelength matching method mentioned in Section 2.2.1. The PMN-28% PT had an acoustic impedance of 25.0 MRayls. For acoustic impedance of the load (tissue or water) 1.5 MRayls, the calculated values of acoustic impedance of the two layers were 7.5 MRayls and 2.3 MRayls, respectively. The layer with higher acoustic impedance was placed adjacent to the piezoelectric element. The model was simulated using PiezoCAD at a design frequency of 3.2 MHz. The transducer with a light backing provided a 6 dB transducer bandwidth of over 80%. With the hard backing, the bandwidth was up to 100% for conventional quarter-wavelength matching. With the double acoustic impedance matching layers, the bandwidth reached 130%. The pulse–echo response of the phased array transducer showed a response and frequency spectrum with a center frequency of 4.0 MHz and with the −6 dB bandwidth of 110% as estimated from the equations described in Section 1.4. The two-way insertion loss was –46.5 dB after compensation for attenuation and reflection from the stainless-steel target. A ring down is seen, indicating partially damped vibration. With the combination of double λ/8 matching layers and hard backing, a transducer bandwidth over 100% is realizable. Similarly, Yongfen et al. [391] used a two-layer acoustic matching method to match the acoustic impedance of planar ultrasonic-receiving-array with 16 × 16 elements made of PbTiO_3_ with the acoustic impedance of oil. Tungsten-loaded epoxy backing was used.

Zhou et al. [36] proposed an advanced endoscopic ultrasonic radial array transducer design using 0.7Pb(Mg_1/3_Nb_2/3_)O_3_-0.3PbTiO_3_(PMN–PT) single crystal/epoxy 1–3 composite. The matching layer was designed considering the load due to tissues of lungs, liver, gallbladder, pancreas, and aorta [392,393,394] for endoscopic applications. The transducer array with 64 elements had dimensions 30.08 mm × 11 mm × 0.165 mm (thickness). The resonance of each element was 6.5 MHz. The backing layer was composed of polyether-modified epoxy resin with tungsten powder and micro-bubbles having high acoustic attenuation to reduce the ring-down time of the transducer. The backing layer was highly flexible to be wrapped onto the copper cylinder. The front-face matching layer was designed considering λ/4 criteria proposed by Desilets et al. [71] as given by Equation (30) and was calculated using the acoustic impedance values 12 MRayl for PMN–PT/epoxy 1–3 composite and approximately 1.5 MRayl for load medium (tissue or water). The matching layer 3.9 MRayl was made by mixing low-viscosity epoxy (Epo-Tek 301) with approximately 5 μm alumina powder. The −6 dB bandwidth was 102%. It also exhibited a low two-way insertion loss of −32.3 dB.

**Figure 19:**
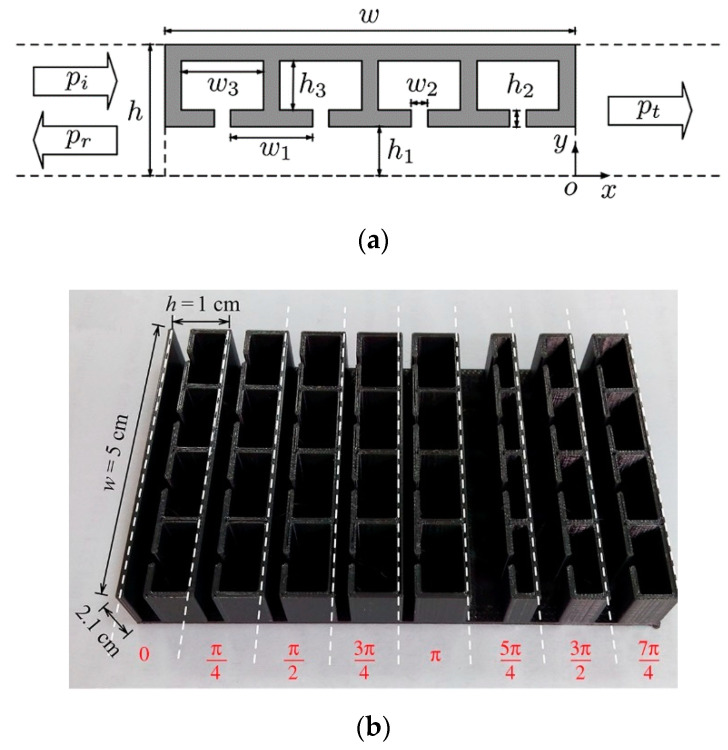

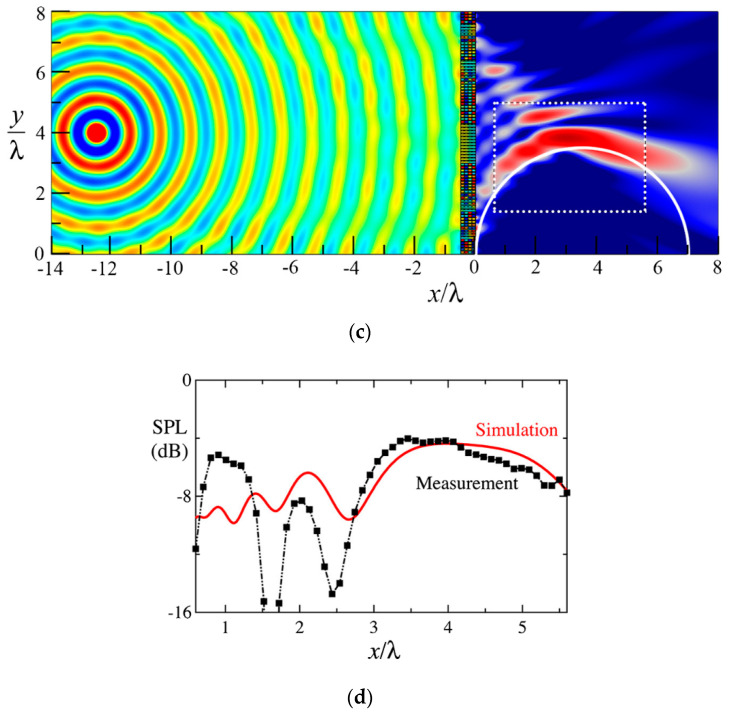
(**a**) Element design showing a hybrid structure consisting of a straight pipe and four Helmholtz resonators. (**b**) Fabricated samples with eight elementary units. (**c**) Simulated sound field where the field at x > 0 is normalized by the maximum value. Comparison of the experimental measurements with numerical simulation along the white-colored trajectory in (**d**). Reprinted with permission from [296] Copyright (2015) by the American Physical Society.

Passive phased array provides local phase delay by steering the wave front from a single source. Apart from shaping the phase and achieving fine spatial resolution, overcoming the local impedance mismatch is required when using acoustic metamaterials [252,253,264,283,293,395,396] and metasurfaces [265,397,398,399,400,401]. Li et al. [296] presented a passive screen stacked up by an array of passive elements forming a hybrid structure. It consisted of a straight pipe of height $h_{1}$ and four Helmholtz resonators (HRs) of height $h_{3}$ with the element dimensions *w* = λ/2 and *h* = λ/10, as shown in Figure 19a. Figure 19b shows the fabricated samples of the eight elementary units to vary the phase from 0 to 2π considering sound waves of resonant wavelength λ = 10 cm at a frequency *f_0_* = 3430 Hz in air. The phase shift produced by such design is given by

(52) $$\emptyset \left(y\right)=-k\left(y-2r\sqrt{\frac{y}{r}}\right)-k\sqrt{{\left(y-\frac{H}{2}\right)}^{2}+L^{2}}-L$$

where L = 120 cm is the distance of the source from the screen, and H = 80 cm is the screen length. A loudspeaker of dimension is 3 × 3 cm^2^ was used to form a wave front, and the sound fields were measured using a ¼ in microphone. The sound field simulated with the white dotted rectangular region for seeking experimental measurements is shown in Figure 19c, whereas Figure 19d shows the experimental measurements. The sound pressure level along the trajectory showed the difference in the near field from imperfect sound absorption by wedge-shaped foams at the metascreen that led to the presence of standing waves in between them.

Brown et al. [402] showed the variation of acoustic impedance of PVDF with temperature from 4.7 MRayl at −40 °C (v = 2630 m/s) to 3.1 MRayl at 80 °C (v = 1730 m/s). The acoustic impedance of the acrylic test blocks 3.1 MRayl matched closely with the PVDF, suggesting that the designers carefully consider the effect of temperature on the acoustic matching properties and finally the transducer design. They used Mason’s model to account for the change in the properties such as dielectric and mechanical losses with temperature. The temperature dependence becomes more important in medical ultrasound where the transducers are continuously operated for imaging and therapeutic applications. Brown [160] has also presented the use of Mylar^TM^ material as matching and protective layers for ultrasonic transducers. Suggestions for design consideration considering various other materials for acoustic matching have been given.

### 4.5. High-Temperature Ultrasound

Ultrasonic transducers for applications at high temperatures have been widely employed within nuclear power industries to inspect steel components at temperatures up to 400 °C. Automotive, aeroengine industries, and materials research use high-temperature transducers [403]. Ultrasonic monitoring is often used to characterize advanced materials during manufacturing at elevated temperatures and during nondestructive evaluation. Ultrasound Doppler velocimetry is another application where hot melts flows are monitored. High temperatures liquid metals inside modern reactors cooled by liquid metals such as Pb/Bi alloy [227,404,405] have wetting limitations and corrosive environment, posing difficulties for the ultrasonic measurements. Space exploration applications of piezoelectric transducers represent several extreme environments with combinations of high temperatures (460 °C), high pressure (9 MPa), and corrosiveness [406,407].

Materials such as polyamides withstand extremely high temperatures while maintaining good ultrasonic properties. They are suitable for the design of acoustic impedance-matching layers for high-temperature transducer applications. Crystals backings and matching layers must be tested, and if necessary, passive cooling is achieved by transducer housings for continuous or long-term operation [226]. A prototype ultrasonic probe proposed by Mrasek et al. [408] involves an inconel-600 λ/2 membrane (approximately 0.6 mm) closing the front of austenite housing with matching acoustic impedance at 800 °C. Several piezoelectric materials serve as active elements for high-temperature ultrasound such as modified bismuth titanate [409], lithium niobate LiNbO_3_ single crystal for temperature up to 1000 °C [410] and Z-cut lithium niobite crystal pillars embedded in a matrix of alumina cement Al_2_O_3_ forming 1–3 connectivity composite materials for operating temperatures above 400 °C [411]. These materials require the development of acoustic impedance materials for their efficient use in high-temperature applications.

Amini et al. [412] introduced a porous ceramic backing layer to operate at 700 °C for broadband ultrasonic transducer applications. A 36° Y-cut lithium niobate (LiNbO_3_) single crystal was used as the piezoelectric element. Zirconia-based backing layer was optimized for the acoustic impedance and attenuation by appropriate selection of its porosity and pore size. Brazing alloy with high temperature and chemical stability was used to bond the transducer layers.

### 4.6. Acoustic Filters

**Figure 20:**
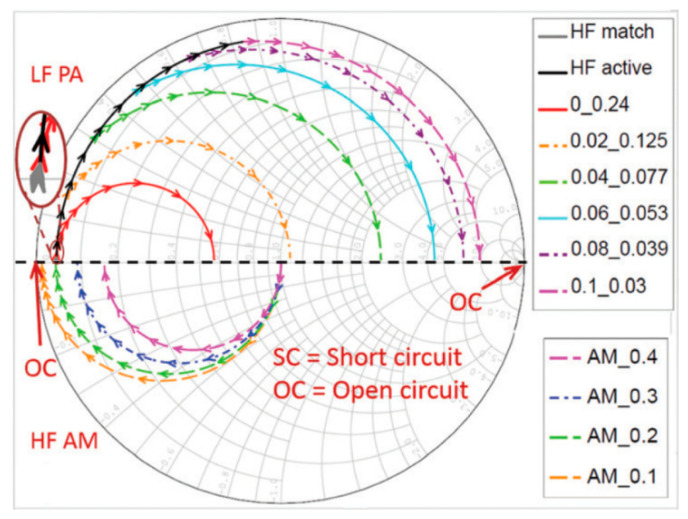
Acoustic filter design calculation using a Smith chart showing the loci of reflection coefficient normalized to the impedance of the piezoelectric material. The numbers followed by *AM* indicate relative impedance. Reprinted from [190], with the permission of AIP Publishing.

**Figure 21:**
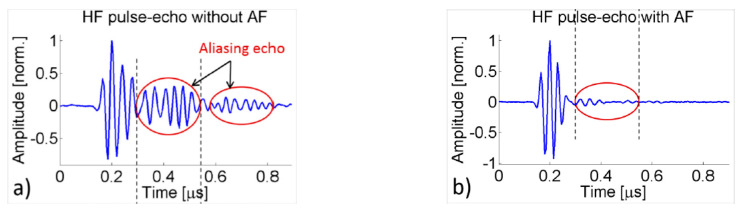
High-frequency pulse–echo response (**a**) without *AF* layer and (**b**) with *AF* layer. Reprinted from [190], with the permission of AIP Publishing.

The analog filters are used as devices in multi-frequency microwave [413], optical [414], and recently mechanical [415] wave control systems. Multi-frequency mechanical or acoustic systems comprise acoustic filters made of polarization patterned piezoelectric solids or periodic structures (phononic crystals) [416,417,418,419] and acoustic diodes and switches (combined periodic and nonlinear systems) [396,420,421,422,423,424,425]. Spatially asymmetric wave propagation using dual-frequency ultrasound transducers [426] favors super-harmonic microscopy [427]. Dual-frequency ultrasound transducer transmits a fundamental wave that blocks its backward propagation. Ma et al. [190] estimated the insertion loss of an *AF* layer as a transmission line using the Smith chart for low-frequency (upper half) and high-frequency (lower half) ranges. The Smith chart represents the loci of the reflection coefficient from the center. As indicated in Figure 20 at high frequency, the matched condition $Z_{H3}$ (center) shifts to free-moving condition $Z_{H2}$. At $l=\lambda_{HF}/4$, the reflection coefficient is maximum with a zero phase yielding very high insertion loss. The AWR software further showed an insertion loss of 10–20 dB with less than 1 dB propagation loss. Experiments performed with $f_{HF}$ = 30 MHz and $f_{LF}$ = 3.5 MHz. Active materials made of PMN-0.33PT had $Z_{HA}$ = $Z_{LA}$ = 36.8 MRayl. Alumina powder mixed with epoxy $Z_{AF}$ = 5.53 MRayl served as an *AF* material. Parylene C $Z_{HM}$ = 3.16 MRayl served as an *HF* matching layer. The thickness was selected such that $L_{AF}$ = 0.25$\lambda_{HF}$ and $L_{HA}$ = 0.5$\lambda_{HF}$. The backing layer chosen as plastic Z = 3.0 MRayl had no effect on performance. The operation of the transducer in pulse–echo mode (in a water bath) showed the aliasing echo as shown in Figure 21 when the *AF* matching layer was absent. In the presence of an *AF* layer between *HF* and *LF* elements, the aliasing echoes were suppressed.

## 5. Acoustic Matching for Specific Applications

### 5.1. Biomedical Applications

The matching of an ultra high-frequency (>100 MHz) ultrasound probe made of high-impedance material with biological tissue and liquid such as water or coupling gel requires a matching layer [387]. For strong acoustic coupling, the transducer’s acoustic impedance should be closely matched to that of body tissue (1.5 MRayls). This also minimizes the reflection from the transducer/skin interface, leading to a low insertion loss. Matching layers assist in the realization of acoustic coupling with the tissue.

#### 5.1.1. Medical Imaging

The reverberations in the medical ultrasound transducers that cause multiple copies of signatures represent as false copies of anatomical structure and degrade the image quality. This paves a way for potential misdiagnosis [1,2,3]. Bertocci et al. [428] presented a method to characterize the reverberations in medical ultrasound transducers. The modeling and simulation of reverberations presented by Kochanski et al. [429] aim in the development of fault detection in medical ultrasound transducers due to reverberations. Identification [430], reverberation cancellation, and reverberation suppression [431,432] are among other works that have reported the complications that arise due to acoustic impedance mismatch. Lee et al. [433] developed a dual-frequency oblong-shaped focused probe for intravascular ultrasound-based tissue harmonic imaging. The first acoustic matching layer had 2.0–3.5 μm silver particles mixed with matrix 7.334 MRayl and had a thickness of 14 μm for the outer elements and 6 μm for the central element. A layer of Parylene C 2.59 MRayl served as protection and electrical shielding layer with a thickness of 7 μm and served as the second matching layer for the 70-MHz center element. The thickness was determined using the PiezoCAD software package (Sonic Concept, Woodinville, WA, USA) through its simulation capabilities. Experiments showed a −6 dB fractional bandwidth to be 50% at 33 MHz in the pulse–echo mode.

#### 5.1.2. Thermal Therapy

Thermal therapy using ultrasound has been used for the treatment of tumors. High-intensity focused ultrasound is a thermal noninvasive treatment methodology alternative to open surgery or chemotherapy for the treatment of non-superficial tumor. Ultrasound used for imaging has a very high frequency of operation where the transducer can be used at high intensities suitable for thermal therapy. Thus, dual-frequency or broadband transducers are preferred instead of using discrete frequency multiple transducers for imaging and thermal therapy. Conventional ultrasound-based probes utilizing PZT4 or PZT8 ($Z_{Piezo}$ ≅ 35 MRayl) ceramics have a narrow bandwidth of 6% but good efficiency of 93%. Chopra et al. [434] noted that the addition of a matching layer (Z = ~7.3 MRayl) based on the quarter-wavelength criteria $\sqrt{Z_{Tissue}Z_{Piezo}}$ and tissue impedance $Z_{Tissue}$ = 1.5225 MRayl, in the absence of a backing layer, gives a broad bandwidth of 64% but with a reduced efficiency of 10%. Further increasing the acoustic impedance of the matching layer nearing the acoustic impedance of piezo ceramic produced two well-spaced frequencies with a high efficiency of 88% and narrow bandwidth. The separation of frequencies served well for ultrasound thermal therapy with narrow bandwidth continuous wave operation and narrow bandwidth high-frequency imaging.

A piezocomposite transducer can transmit acoustic intensity in order of 10 W/cm^2^ with 60% efficiency for 60 s, which is suitable for therapeutic applications [126]. A piezocomposite material possessing around 8–12 MRayl allows good energy transfer with a wide bandwidth and with efficient coupling to water or tissue [435]. With the flexibility of the polymer phase with the bending capability of embedded piezoelectric fibers or rods or particles, the fabrication of transducers with the concave or convex surface is easily realized. However, the low mechanical quality factor of piezocomposites results in heat generation alongside high-power or high-intensity acoustic transmission. The low thermal conductivity of the polymer phase adds to the problem of thermal dissipation, giving rise to heat accumulation and the depolarization of piezoelectric material due to heat. Acoustic impedance matching with a proper choice of piezoelectric material can provide better designs.

Acoustic pressure that is high enough to exceed the cavitation threshold increases the temperature, and this leads to shortening of the thermal therapy treatment duration [436]. Cavitation produces mechanical and chemical effects such as sonoluminescence and sonochemical reactions in tissues for potential therapeutic applications [437,438,439,440,441,442], lithotripsy [443], and histotripsy [444].

**Figure 22:**
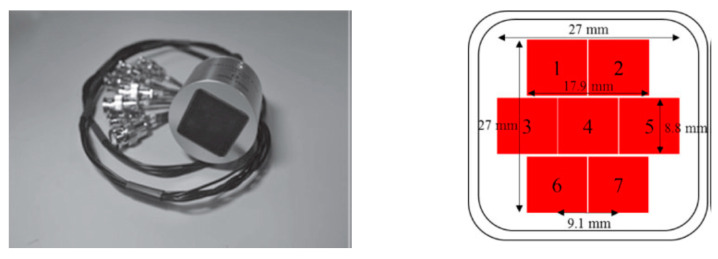
(**a**) Heavy matching layer transducer and (**b**) its internal configuration. Reproduced from [448] with permission from Copyright (2016) The Japan Society of Applied Physics.

**Figure 23:**
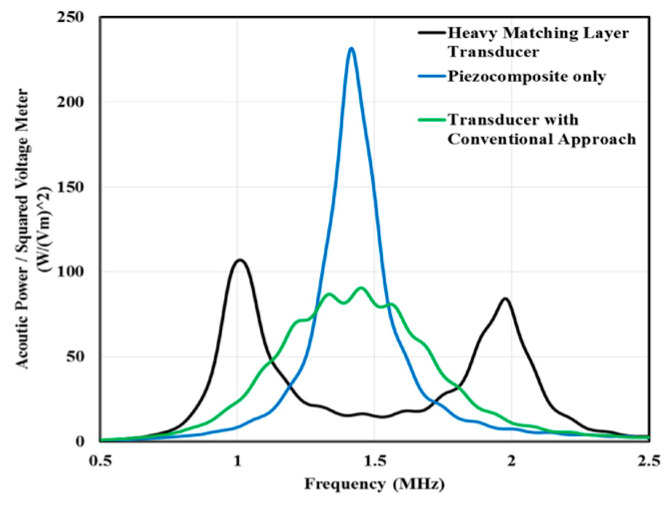
Comparison of transducer output with a heavy matching layer, without a heavy matching layer, and with a conventional approach. Reproduced from [448] with permission from Copyright (2016) The Japan Society of Applied Physics.

Usage of the second harmonic in the diagnostic ultrasound field is widely used, especially in low-frequency medical application [445]. Superimposing the second harmonic upon the fundamental harmonic increases the cavitation bubbles when compared to only single-frequency waves [446,447]. Zaini et al. [448] used high impedance-matching layers for a transducer consisting of seven electrically independent square elements. It was designed for a frequency of 2 MHz for generating the second harmonic. The width and height of each element was 8.8 mm, as shown in Figure 22. The thickness ratio of the piezocomposite and heavy matching layer was modified with initial thickness values of a piezocomposite and heavy matching layer of 0.95 mm and 0.46 mm, respectively. By increasing the thickness of the heavy matching layer to 0.506 mm and decreasing the thickness of the piezocomposite to 0.903 mm, the amplitude of the fundamental component at 1 MHz was decreased, and the second harmonic at 2 MHz was increased. The fundamental and second harmonic amplitudes can be tuned by varying the thickness ratio of the piezocomposite and the heavy matching layer. The simulation and experimental results with a heavy matching layer produced both the fundamental and second harmonic, as shown in Figure 23.

#### 5.1.3. Dental

Piezoelectric vibrators with kilohertz-frequency vibrating tips have been used for scaling teeth, and limited developments have been realized for the diagnostic imaging of tooth when compared to medical diagnostics [449]. Imaging has been realized involving surrounding organs such as tongue, major salivary gland, lymph nodes, facial, and neck muscles to detect diseases such as carcinomas, periapical lesions, and temporomandibular joint disorders [450]. The reason for limited advancements in the imaging of tooth and the immediate surrounding areas is the anisotropic material properties [451], causing drastic variation in the velocity measurements depending on the position and direction [449]. The geometry of the tooth adds to the prevailing complexities, making the imaging erroneous. The properties of the tooth and its surrounding tissue are listed in Table 6.

#### 5.1.4. Opthalmic

Ultrasound imaging provides noninvasive cross-sectional images of soft tissues [178] such as eye and blood vessels with high-frequency transducers, as high resolution is needed. The image resolution requirement has prompted intensive studies as it assists in diagnosing disease and tissue damage or abnormality at very early stages. Very high (approximately 100 MHz) [128] to ultra-high (100–300 MHz) [172] frequency designs provide such resolutions. Design, fabrication, and characterization have been proposed by Zhang et al. [454] to develop a press-focused LiNbO_3_ transducer. It consisted of a 36° Y cut LNO single crystal with one matching layer to meet the very high-frequency requirement. A simulation software PiezoCAD (Sonic Concepts, Woodinville, WA) based on the KLM model was used to design the transducer aperture size and optimize the thickness. Large bandwidth (92% at −6 dB) was achieved at a frequency of 75 MHz for a pulse–echo response. The scanning of pig eyes showed fine structures showing a lateral resolution of 110 µm and an axial resolution of $R_{axial}$ = 13.09 µm. These resolutions are estimated [173] by

(53) $$R_{axial}=\frac{\lambda }{2\;BW}=\frac{v}{2f_{c}\;BW}$$

(54) $$R_{lateral}=\frac{\lambda F^{\prime }vF}{f_{c}}$$

where v is the speed of sound, $f_{c}$ is the center frequency, BW is the bandwidth, $F^{\prime }$ is the ratio of focal distance to aperture dimension, and λ is the wavelength.

#### 5.1.5. Implants

Wireless implantable devices are popular since the 1970s when they were introduced based on the inductive powering principle [455]. However, these devices operate with transmitter and receiver coils placed less than 4–10 mm apart [456]. Deeply placed implants cannot operate based on the inductive principle. Ultrasonic powering has overcome this challenge [457], as ultrasonic waves can propagate in the body tissues with small dissipation, even in the frequency range of 1–10 MHz. Devices of size 10–50 mm have been reported with the capability to power and operate at the tissue depths of 5–10 cm [458,459]. Omnidirectional ultrasonic transducers producing high intensity focused ultrasound provides proper reception of signals overcoming reflections at interfaces such as fat/muscle or bone/tissue. High-intensity ultrasound is harmful due to thermal and other mechanical effects [460,461,462]. The treatment of hyperthermia with the intensity of 1–1000 W/cm^2^ for a few seconds [463] and imaging applications involving the Food and Drug Administration (FDA), USA-approved intensity of 720 mW/cm^2^ [464,465] have been used so far to avoid the thermal bioeffect, which is a major hazard. Song et al. [155] presented an ultrasonic powered implantable device located 10 cm away from the transmitter. Assuming the operation at a resonant frequency, they modeled the power transfer using a Thevenin equivalent model [466], where the power output available at the receiver end $P_{OUT}$ is proportional to the input power $P_{IN}$ as per the relation

(55) $$\eta =\frac{P_{IN}}{P_{OUT}}=\frac{{\left(\alpha T\phi_{R}\frac{Z_{L}}{Z_{L}+Z_{OUT}}\right)}^{2}}{C_{T}C_{R}V_{IN}^{2}}\;,$$

where $P_{IN}=C_{T}V_{IN}^{2}f_{r}/2$, $f_{r}=v/\left(2d\right)$ is the resonant frequency of operation, v is the acoustic velocity in the piezoelectric material, d is the piezoelectric material thickness, $T\approx 2Z_{R}v$ is the ultrasonic pressure, $\alpha =e^{-2\mu d_{I}}$ is the tissue attenuation, μ is the attenuation coefficient, $d_{I}$ is the implant depth in the tissue, $V_{IN}$ is the input source voltage, $Z_{L}$ is the electric load impedance at the receiver due to implanting conditions, and $Z_{OUT}$ is the output impedance due to the receiver capacitance $C_{R}$. The capacitance of the transmitter $C_{T}$ and receiver $C_{R}$ is modeled as

(56) $$C=\frac{\epsilon_{33}^{S}\epsilon_{0}A}{d},$$

where $\epsilon_{33}^{S}$ is the clamped relative complex permittivity. The $V_{IN}$ is fixed to limit T to radiate power within FDA limits (720 mW/cm^2^). This condition is used further to design the impedance of the matching layer based on a single quarter layer matching using a transmission line model as given by Equation (30) and as described in Section 2.2.1. The electric-to-mechanical energy conversion or vice versa is conveniently represented by the transformer with a turn ratio of Φ:1 described by the relation

(57) $$\Phi =\frac{1}{2\frac{h_{33}}{\omega Z_{0}}\sin\left(\frac{\beta t}{2}\right)}\;,$$

where β=2π/λ, λ is the wavelength and $h_{33}=k_{t}\sqrt{C_{33}^{D}/\left(\epsilon_{33}^{S}\epsilon_{0}\right)}$ is the piezoelectric pressure constant related to electromechanical coupling coefficient $k_{t}$. The *I-V* characteristics from experiments revealed the availability of 10 mW/cm^2^ acoustic power at the receiver placed 20 cm far from the source as per the far-field criteria [467] $N=\frac{D^{2}}{4\lambda },$ where N is the far-field distance and D is the dimension of the transducer. The efficiencies of 2.7% was seen in 2 × 4 × 2 mm^3^ receivers. The quarter-layer impedance-matching layer for the transmitter was made of 40% by wt iron oxide-loaded epoxy having around 6.75 MRayl acoustic impedance [237,468].

### 5.2. Cavitation

The acoustic cavitation phenomenon has been employed in many applications such as high-power ultrasound [469,470,471,472], inertial cavitation erosion [473] and dispersion, surface cleaning, and degreasing [474]. Quantifying acoustic cavitation is helpful to determine physical erosion and chemical species produced by bubble collapse [475,476]. A lack of suitable measurement sensors for determining noninertial (stable) cavitation or inertial (transient) cavitation has inhibited the development of high-power measurement systems [470,475,477]. Chemiluminescent or sonoluminescent cavitation detection and measurement techniques have high spatial and temporal resolution but are limited to transparent media [477]. Features of the acoustic emission spectra from cavitation can be modeled from theoretical analysis including sharp harmonics and ultraharmonics arising from the nonlinear motion of the bubble oscillator and broadband white-noise output due to violent inertial cavitation [478]. White noise from cavitation is measured using miniature piezo-electric detectors (hydrophones) [479].

Zeqiri et al. [480] presented an ultrasonic cavitation sensor design specifically for monitoring acoustic emissions generated by small microbubbles. A 110 µm layer of piezoelectrically active film enabled acoustic emissions measurements beyond 10 MHz. The absorber was designed to shield the outside vibrations by increasing the density of the absorbing material to match the acoustic impedance to water at kilohertz frequencies. Polyurethane layers containing Expancel concentrations 2.7–25 vol.% with thickness from 3 mm to 4 mm were fabricated. Acoustic transmission property was determined as discussed in [481], and the transmission loss as a function of the concentration of Expancel microspheres was obtained. The transmission loss of the material was dependent on the air fraction values and was found to be as high as 90 dB/(cm MHz). The speed of sound propagation determined at 1 MHz rapidly decreased to 960 m/s at 25% Expancel loading.

## 6. Conclusions

This review article presented the theory of smart materials in the context of their use in sensors and transducers considering various aspects of acoustic impedance-matching layer design. The important aspects of the material properties and vibration modes of smart materials to be considered while designing the acoustic matching layers has been discussed. Conventional materials and special materials used to design active and passive acoustic impedance matching layers for specific applications and transducer type has been presented. Acoustic impedance matching techniques use several models, methods, and tools for the efficient design of transducers and were discussed considering the limitations or requirements posed by transducer type. Another section was devoted to the discussion of acoustic impedance-matching techniques considering the requirements posed by a specific application, especially in the biomedical field for frequencies ranging from ultra-high (>100 MHz) to microwave (20 GHz). Apart from composite materials, metamaterials hold a promising future in designing both active elements and passive layers to achieve better acoustic impedance matching that dramatically increases the transducer efficiency, bandwidth, or both.

## Figures and Tables

**Table 1:** Electric and acoustic properties of commonly used bulk piezoceramic materials for transducer applications. PMN–PT: lead magnesium niobate-lead titanate, PZT: lead zirconate titanate.

	Piezoceramic Lead Zirconate Titanate (Synthetic) [67,75,77,85,101,102]						Lead-Free Piezoceramics (Synthetic)			
Properties	PZT4	PZT5A	PZT5H	PZT6B	PZT7A	PZT8	BaTiO_3_ [85]	KNaNb_2_O_6_ [85]	LiNbO_3_ [85,103]	PMN–PT (33% PT) [67]
Curie Point (°C)	325	365	200	350	350	300	115	420	1150	130
$d_{33}$ (pC/N)	285	374	593	71	153	225	190	127	6	5500
$d_{31}$ (pC/N)	−122	−171	−265	−27	−60	−97	−78	−51	-	
$d_{15}$ (pC/N)	495	585		130	360	330	260	306	69	
$\epsilon_{33}^{T}$	1300	1700		460	425	1000	1700	495	25	
$k_{33}$	0.70	0.71	0.75	0.37	0.67	0.64	0.50	0.60	0.23	0.94
$k_{31}$	−0.33	−0.34	0.36	−0.15	−0.30	−0.30	0.21	−0.27	-	
$k_{15}$	0.71	0.69		0.38	0.68	0.55	0.48	−0.46	0.60	
$g_{33}$ (Vm-N)	0.025	0.0166	0.0125							
$S_{33}^{E}$ (10^−12^ m^2^/N)	15.5	18.8		9.35	13.9	13.5	-	-	-	
$S_{11}^{E}$ (10^−12^ m^2^/N)	12.3	16.4		9.0	10.7	11.5	-	-	-	
$\epsilon_{33}^{T}/\epsilon_{0}$			1470				-	-	39	680–800
$\epsilon_{33}^{S}S/\epsilon_{0}$	635	830	1470	385	235	580	-	-	-	680–800
Mechanical Q		75	65		32					
ρ (kg/m^3^)	7700	7700	7500				5400		4640	8060
c (m/s)	4600		4580				4000		7640	4610
$Z_{ACO}$ (MRayl)	36.15	30.00	34.35	30.00	32.00		30		34	37.15

^T^ Parameters measured in stress free conditions, ^E^ parameters measured in short circuit conditions, $Z_{ACO}$—acoustic impedance, $S_{ij}$ compliance constants, ρ—density, c—velocity of sound in media, 1 Rayl = 1 N-s/m^3^ = 1 kg m^−2^ s^−1^ [101].

**Table 2:** Properties of bulk piezopolymers.

Properties	PVDF [74,113,117,118,119,121]	P(VDF-TrFE) [120,121]	Parylene-C [121]	Amorphous polyimide PI-(β-CN) APB/OPDA [121]
Curie Point (°C)	160	115	-	-
Max temp (°C)	90	100	-	220
$d_{33}$ (pC/N)	−13.6	−33.5	2.0	5.3–16.5
$d_{31}$ (pC/N)	5.3	10.7	-	-
$d_{32}$ (pC/N)	1.5	10.1	-	-
$d_{15}$ (pC/N)	−27	−36.3	-	-
$g_{33}$ (Vm-N)	−0.32	−0.38	-	-
$k_{33}$ (percent)	4.4	6.2	0.02	0.048–0.15
$k_{31}$ (percent)	3	-	-	-
k [100]	0.15–0.2	0.3	-	-
Y (GPa)	2.5–3.2	1.1–3	2.8	2–3
$S_{11}^{D}$ (GN/m^2^)	3.70	3.01	-	-
$S_{22}^{D}$ (GN/m^2^)	3.20	2.99	-	-
$C_{33}^{D}$ (GN/m^2^)	1.51	3.33	-	-
$S_{66}^{D}$ (GN/m^2^)	0.7	0.69	-	-
$\epsilon_{r}$	12	12	3.15	4
$\epsilon_{11}^{T}/\epsilon_{0}$	6.9	7.40	-	-
$\epsilon_{22}^{T}/\epsilon_{0}$	8.6	7.95	-	-
$\epsilon_{33}^{T}/\epsilon_{0}$	7.6	7.90	-	-
Tan $\delta_{e}$	0.018	0.018	-	0.01
Tan $\delta_{m}$	0.05	0.05	0.06	0.06
Q [100]	10	25	-	-
ρ (kg/m^3^)	1760	1880	1100–1290	1420
c (m/s)	2260	2400	2350	-
$Z_{ACO}$ (MRayl)	20	4.51	2.58	-

Y is the Young’s modulus, PVDF is polyvinylidene fluoride, Tan $\delta_{e}$ is the dielectric loss, Tan $\delta_{m}$ is the mechanical loss, Q is the mechanical quality factor, and k is the electromechanical coupling coefficient.

**Table 3:** Properties of piezocomposites with different types of piezoelectric fillers such as fibers and particles. MFC: macro fiber composite.

Properties	MFC [121,148,149]	PMN-0.29PT/Epoxy [121,150]	ZNo/SU-8 20%	BURPS [151]	Macrovoid [85]	Perforated [152]	Honeycomb [85]	Perforated [152]	Weave [85]	Laminate [85,153]
Connectivity	1-3	1-3	0-3	3-3	3-0	0-3	3-1	3-2	2-3	2-2
Filler	PZT	PMN-0.29PT	ZNo	PZT(50%)	Void	PZT	PZT	PZT	-	PZT (15–30%)
Matrix	Epoxy	Epoxy	SU-8 20%	Epoxy	PZT	Silicone rubber	Polymer	Epoxy	-	Epoxy
Max use temp (°C)	130	130	180	-	-	-	-	-	-	-
Elastic Constants (GPa)	$E_{1}\;$ = 30.34; $E_{3}$ = 15.86; $G_{13}$ = 5.52; $\upsilon_{13}$ = 0.31	-	-	-	-	-	-	-	-	-
$d_{h}$ (pC/N)	714	-	-	120	150	28.3	100	329	80	50
$g_{h}$ (pC/N)	-	-	-	27	30	32	30	128	65	16.6
$d_{33}$ (pC/N)	374	1200	5–8	160	-	-	-	290	-	410 to 440
$d_{31}$ (pC/N)	170	-	5	-	-	-	-	-	-	−188 to −200
$k_{33}$ (percent)	0.53	0.9	0.07–0.12	-	-	-	-	-	-	-
$k_{31}$ (percent)	0.34	-	0.07	-	-	-	-	-	-	-
$\epsilon^{T}$	850	2000	7	500	560	100	400	290	140	340
ρ (kg/m^3^)	5440–7500	5000	-	-	-	-	-	-	-	-

**Table 4:** Acoustic properties of typical piezoelectric transducer materials and media [154,155,156,157,158,159,160].

Material	Typical Function	ρ (kg/m^3^)	c (m/s)	$Z_{ACO}=\rho c\;(\mathrm{MRayl})$	Attenuation or Loss
LiNbO_3_ Crystal	Active element	7360	4688	34.5	-
Quartz	Active element	2650	5740	15.2	-
PZT5A [100,161]	Active element	7750	4350	33.7	0.02 (dB/cm.MHz)
PZT4	Active element	-	-	36.15	-
PMN–PT	Active element	8100	3950	32.0	-
Parylene	Matching layer	2350	1100	2.58	-
Gold	Matching layer	3240	19700	63.8	-
Aluminium	Matching layer	2700	6320	17	-
Steel	Matching layer	7700	5900	45	-
Glass	Matching layer	3000	5000	15	-
Perspex	Matching layer	1180	2730	3.2	-
Polystrene	Matching layer	1060	2350	2.5	-
AAO–epoxy	Matching layer	2745	3460	9.5	-
HDPE	Matching layer	951	2339	2.22	12.41 (Np/m)
Syntactic foam	Matching layer	704	2486	1.75	41.3 (Np/m)
2 µm Al_2_O_3_/Epotek 301	Matching layer	2300	2800	6.4	1.68 (dB/mm at 3.2 MHz)
Teflon	Matching layer	2200	1390	2.97	-
Polycarbonate	Matching layer	1220	2300	2.75	-
Acrylonitrile-butadiene-styrene	Matching layer	1060	2510	2.68	-
Polypropylene	Matching layer	920	2740	2.4	-
Polysulfone	Matching layer	1240	2240	2.78	-
Mylar	Matching layer	1380	2540	3.00	-
Epotek 301	Lens/Epoxy	1048	2640	2.8	1.04 (dB/mm at 3.2 MHz)
E-Solder 3022	Conductive backing	1850	3200	5.92	11.8 (dB/mm at 3.2 MHz)
Tungsten powder/µ bubbles/Epotek 301	Backing layer	3570	1820	6.5	-
5 µm Tungsten powder/micro bubbles/Epotek 301	Heavy backing layer	8925	1800	16.0	16 (dB/mm at 3.2 MHz)
Epoxy EPO-TEK 301	Insulation	2650	1150	3.05	-
Water	Media	997	1450–1498	1.445–1.5; 1.48 [155]	0.002 (dB/cm.MHz)
Air (Rayl)	Media	1.225	343	0.000420	1.64 (dB/cm.MHz)
Tissue (Blood)	Media	-	-	1.66	0.15 (dB/cm.MHz)
Tissue (Fat)	Media	-	-	1.38	0.6 (dB/cm.MHz)
Tissue (Bone)	Media	-	-	7.75	2–15 (dB/cm.MHz)
Tissue (Skin)	Media	-	-	1.99	9.2 (dB/cm.MHz)
Concrete [162]	Media	-	-	8.36–11.3	-

HDPE is high density poly ethylene, AAO is anodic aluminum oxide and EPO-TEK is a registered trademark of Epoxy Technology, Inc., and is an adhesive.

**Table 5:** Comparison of acoustic properties of passive composite and nanocomposite matching layer and their performance.

Transducer Type	Active Element	Acoustic Load	Matching Layer Material	$\mathrm{Matching}\;Z_{ACO}\;\;(\mathrm{MRayl})$	Bandwidth	Loss	Ref
Ultrasonic imaging (12 MHz)	Lead zirconate titanate piezo ceramic	Human tissue	AAO–epoxy 1-3 composite	9.1 and 2.4 for 2 layers	68% (−6 dB)	2-way insertion −22.7 dB	[157]
Ultrasonic imaging (40 MHz)	LiNbO_3_	Human tissue	alumina/polymer	2.8 to 5.1 for single layer	35% (−6 dB)	15 dB/mm	[237]
Medical ultrasound (100 MHz)	Zinc oxide layer	Water	cerium oxide/polymer	4.0 to 7.0 for single layer	(signal enhanced by 100%)	0.5 dB/µm	[238]
Medical ultrasound (>50 MHz)	PZT piezoceramics	Human tissue	Silicon oxide colloidal/polymer	4.4 to 5.8 for single layer	-	-	[239]
Ultrasonic imaging (1 GHz)	Silicon	Water	Su8/TiO_2_	3.0 to 6.0	-	0.5 dB/µm	[240]
Medical ultrasound (15 MHz)	PZT piezoceramics	Human tissue	silicon-polymer 1-3 composites	5.54 to 6.32	50% (−6 dB)	-	[241]

**Table 6:** Acoustic properties of typical dental media. $c_{l}$ and $c_{t}$ represent the longitudinal and transverse wave velocity [449,452,453].

Material	$c_{l}\;(m/s)$	$c_{t}\;(m/s)$	ρ (kg/m^3^)	$Z_{ACO}=\rho c\;(\mathrm{MRayl})$
Enamel	6250	3100	3000	18.8
Dentin	3800	1900	2000	7.6
Cementum	3200	-	-	6.5
Dental Pulp	1570	800	1000	1.57
Ligament	1580	-	-	1.7
Gingiva	1540	-	-	1.63
Amalgam	4350	2260	7750	33.7
